# Stability analysis of a multiscale model of cell cycle dynamics coupled with quiescent and proliferating cell populations

**DOI:** 10.1371/journal.pone.0280621

**Published:** 2023-01-20

**Authors:** Iqra Batool, Naim Bajcinca

**Affiliations:** Rheinland-Pfälzische Technische Universität Kaiserslautern-Landau, Mechanical and Process Engineering, Kaiserslautern, Germany; Cardiff University, UNITED KINGDOM

## Abstract

In this paper, we perform a mathematical analysis of our proposed nonlinear, multiscale mathematical model of physiologically structured quiescent and proliferating cell populations at the macroscale and cell-cycle proteins at the microscale. Cell cycle dynamics (microscale) are driven by growth factors derived from the total cell population of quiescent and proliferating cells. Cell-cycle protein concentrations, on the other hand, determine the rates of transition between the two subpopulations. Our model demonstrates the underlying impact of cell cycle dynamics on the evolution of cell population in a tissue. We study the model’s well-posedness, derive steady-state solutions, and find sufficient conditions for the stability of steady-state solutions using semigroup and spectral theory. Finally, we performed numerical simulations to see how the parameters affect the model’s nonlinear dynamics.

## 1 Introduction

One of the cornerstones in understanding human tumor growth is mammalian cell division patterns. Many researchers have been drawn to it, and it has been the subject of extensive research for decades. Most theoretical research works explore the life cycle by utilizing age-structured frameworks. Some examples of age-structured growth models include epidemic [1–3], microscopic virus [4, 5] and and cell population [6–9] models. However, the underlying molecular intricacies of a tissue necessitate a more comprehensive modeling framework comprising special molecular and cellular interactions.

**Fig 1:**
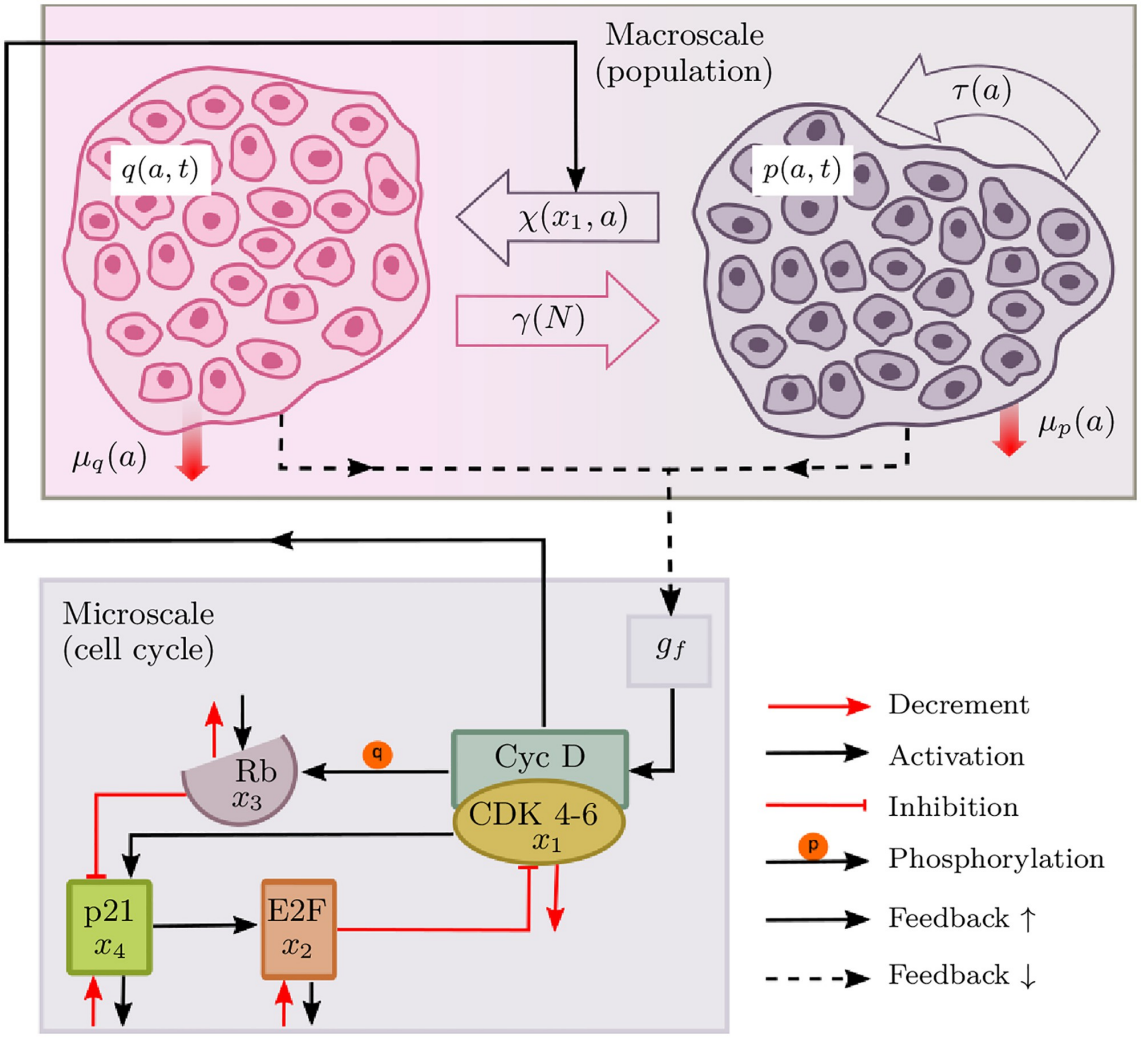
Model schematics. In the macro-scale, two subpopulations are proliferating and quiescent cells with various transition effects given by *χ*, *τ*, *γ*, and *μ* functions. At the bottom, the microscale is represented with all four protein states and their interactions which are explained using legends in the bottom left. The feedback from the macroscale, in the form of growth factors *g*_*f*_, manipulates the cell-cycle (microscale). The feedback loop is closed by the rate *χ* (corresponds to the rate of cells transitioning from proliferating to quiescent phase), determined by the protein dynamics at the microscale.

In any living tissue, the dividing cells can be classified into quiescent and proliferating compartments. Proliferating cells divide by going through various stages in cell-cycle (*G*_1_, *S*, *G*_2_, *M*). Quiescent cells, on the other hand, do not grow or proliferate; instead, they move from the proliferative compartment to the *G*_0_ phase and remain there until differentiation or apoptosis. For tissue homeostasis to be preserved, cells must be able to switch between the quiescent and proliferative phases. However, the transitioning between the two compartments relies on signaling molecules, which are known as growth or anti-growth factors [10]. Proliferating cells grow within a tumor cell population until the tumor is active and malignant. Besides, the total number of cells, i.e., in both quiescent and proliferating cell populations, remains stable (on average) to preserve homeostasis; therefore, the size of the proliferative compartment in a healthy cell population remains confined. The schematics of a multiscale modeling framework employed in this paper is shown below in Fig 1.

This work primarily focuses on formulating a model of the cell population (in both proliferative and quiescent compartments) and analyzing its dynamics concerning the behavior of cell-cycle proteins. Age-structured models, as previously indicated, have been widely employed in this direction. These include models investigating cell population only in quiescent phase [9], cell population only in proliferating phase [11, 12]. Finally, cell population dynamics involving both quiescent and proliferating phases [8, 13–17]. Nevertheless, the influence of molecular interactions at the subcellular level on balance between proliferative and quiescent phases has not been studied. The main objective of this paper is to formulate a multiscale model by employing mathematical tools which can also encompass the heterogeneity of a complex system lying at the sub-cellular level. Therefore, we primarily focus on two predominant scales, i.e., macroscale (population dynamics) and microscale (cell-cycle dynamics), and define the coupling between these two time and age varying scales. Age refers to the time elapsed since last division, [12, 18]. Note that in addition to the physical time variable (denoted by *t*), age-structured models introduce the age variable (denoted as *a*) which has rather a physiological character. The concept of “cell age” characterizes the biological variability within a proliferating cell population. Partial differential equations (PDEs) are used to simulate cell populations in the quiescent and proliferative stages at the macroscale. While ordinary differential equations are used to predict sub-cellular protein interactions related to cell-cycle dynamics (ODEs). Finally, through feedback in both directions, the two scales are connected. As mentioned earlier, proliferating cells represent a complete cycle of cell division (*G*_1_,*S*,*G*_2_,*M*). Cells in the early proliferating phase, known as G_1_, can transition to the quiescent phase till they reach the restriction point (R). However, depending on the concentration of *G*_1_ phase cyclin protein (*x*_1_), the cells transit to *S* phase from late G_1_ phase. It is also clear that restriction point (R) splits the cells in the G_1_-phase in two parts such that the cells become quiescent before R but can no longer avoid division once R is passed, [19, 20]. In quiescent phase, cells do not divide or grow, but they continue to perform their other cellular functions. A bidirectional cell transitioning between quiescence and proliferation phases plays an essential role in tissue homeostasis, and it is regulated by extracellular environmental conditions [10]. In tumoral tissue, the balance in the bidirectional transition is disturbed, and cells may grow unconditionally [21]. Recent experiments have also revealed that cyclins are the most significant regulatory molecules for changes in cell-cycle phase, [22]. As a result, we use a crucial aspect in the dynamics of cell-cycle (i.e., from *G*_1_−*S* phase transition) to predict the evolution of a transitional balance between quiescent and proliferating subpopulations, that is essential to maintain homeostasis.

A variety of proteins are expressed at the microscale, which play an essential role in the sequential transition between different phases of cell-cycle. The complex network of protein interactions in the cell-cycle has been mathematically described using ODEs and simulated by several authors, including [23–28] and references therein. However, for simplicity, we consider only four proteins (i.e., Cyclin D-CDK4/6, p21, E2F, and Rb) from the network of proteins which participate in the cell-cycle dynamics. These proteins are chosen because they are primarily engaged in Cyclin D’s activity and the progression of cells to the *S* from *G*_1_ phase. The motivation stems from experimental results, which have shown that Cyclin D regulates the transition between the *G*_0_ and *G*_1_ phase, see [29–31]. Furthermore, when Cyclin D is over-expressed, cells in the proliferative phase commit to cell division, and when Cyclin D is under-expressed, cells enter a quiescent phase. It should be noted that these molecular interactions are assumed to occur in a fast growing population of cells and not in a single cell. Moreover, we assume averaged concentrations of these proteins in proliferating and quiescent cell subpopulations without considering cell to cell variability. In the sequel, we provide the biological relevance of cell-cycle proteins. The advancement in the cell-cycle is regulated by cyclin proteins (structural protein) and their cyclin-dependent kinase (CDK) inhibitors. There is a specific Cyclin/CDK complex for every phase in the cell-cycle. When micro-environment of a cell has enough growth signals, it initiates a cell-cycle that spans the activities of phase-specific complexes of cyclin protein and their catalytic partners CDK. Cyclin D activates during the *G*_1_ phase and is induced merely by growth factors, [32]. When there are no growth factors, the concentration of Cyclin D declines, and the cell does not start the cycle. Growth-factors attach to particular receptors located on the external cytoplasmic membrane of the cell, which activates intra-cellular signaling pathways (i.e., Raf/Map/Ras kinase), which ultimately leads to the synthesis of Cyclin D (see [33–35], for more details). Cyclin D makes an active complex with CDK4/6 with a maximum synthesis rate. This complex can then trigger the activation of transcription factor E2F by phosphorylating its inhibitor retinoblastoma protein Rb. Resultantly, the transcription factor E2F is accumulated and activates the other essential cyclins involved in the cell-cycle.

To summarize, we develop a multiscale model to primarily address the concerns relevant to impairment in cell transitioning between quiescent and proliferating compartments, which results in unlimited tumor growth, and whether Cyclin D is responsible for the deregulation of cells transitioning between quiescent and proliferating compartments.

The layout of the paper is given in the sequel: Section 2 delves into the depth of multiscale mathematical modeling of quiescent and proliferating cell populations associated with cell-cycle dynamics. In Section 3, we first demonstrate the uniqueness and existence of non-negative solutions using semigroup and spectral theory from functional analysis to confirm that the governing model equations are well-posed. Next, in Section 4, we first derive steady-state solutions and then obtain spectral criteria for local stability for steady-state solutions in a way that in the sense that if the growth bound of the linearised semigroup is negative, the steady-state solution is the locally asymptotically stable, and if growth bound is positive, the steady-state solution is unstable. Finally, Sections 5 and 6 contain the discussion about the results and final conclusion of the paper, respectively.

## 2 Mathematical modeling

### 2.1 Age-structured model

The cell populations in quiescent and proliferating compartments are described by transport PDEs (partial differential equations) of nonlinear hyperbolic type, which characterize the density distribution of the cells concerning physiological age *a* and time *t*. In the quiescent phase, the cell density *q*(*a*, *t*) is given by

$$\begin{array}{c} \frac{\partial }{\partial t}q\left(a,t\right)=\chi \left(a,x_{1}\right)p\left(a,t\right)-\left(\gamma \left(N\right)+\mu_{q}\left(a\right)\right)q\left(a,t\right), \end{array}$$ (1)

where the first term *χ*(*a*, *x*_1_)*p*(*a*, *t*) is the inflow from the proliferating cells at the rate *χ*(*a*, *x*_1_), which is further regulated by a microscale variable, namely the age-specific concentration of Cyclin complex *x*_1_. The detail of the microscale variables is presented later in this section. The next term refers to the loss in quiescent cell density caused by either returning to cell division with the rate *γ*(*N*) in the proliferating phase or by cell death as a result of apoptosis (or necrosis), as depicted by death rate *μ*_*q*_(*a*). The total number cell population in both phases is represented by *N*(*t*), which is defined in Eq (3). The cells in the quiescent phase do not age (or in other words, the cells halt their age), therefore in Eq (1), the convection term concerning physiological age *a* is not present. In the proliferating phase, the cell number density represented by *p*(*a*, *t*) reads

$$\begin{array}{c} \frac{\partial }{\partial t}p\left(a,t\right)+\frac{\partial }{\partial a}\left(g\left(a\right)p\left(a,t\right)\right)=\gamma \left(N\right)q\left(a,t\right)-\left(\tau \left(a\right)+\chi \left(a,x_{1}\right)+\mu_{p}\left(a\right)\right)p\left(a,t\right), \end{array}$$ (2)

where *g*(*a*) stands for the rate of evolution of a cell-cycle. The first term on the right *γ*(*N*)*q*(*a*, *t*) denotes the transition from the quiescent to the proliferating cells. The following term *τ*(*a*)*p*(*a*, *t*) symbolizes the number of cells that complete cell division at some age of the proliferating phase, whereas the cells that are moving to the quiescent phase from proliferating phase without having undergone division are given by the term *χ*(*a*, *x*_1_)*p*(*a*, *t*). Finally, the decrement in proliferating cell density because of apoptosis/necrosis is described by the death rate *μ*_*p*_(*a*). The cell population, *N*(*t*), defined as the sum of all cells in the quiescent and proliferating phases across all ages, is given as:

$$\begin{array}{c} N\left(t\right)=\int_{0}^{a^{⋆}}(q(a,t)+p(a,t))da, \end{array}$$ (3)

where *a*^⋆^ is the maximal age of the cells. The initial conditions are defined as:

$$\begin{array}{c} p\left(a,0\right)=p_{0}\left(a\right),\;q\left(a,0\right)=q_{0}\left(a\right),\;\forall a\geq 0. \end{array}$$ (4)

The boundary condition is given as follows:

$$\begin{array}{cc} g\left(0\right)p\left(0,t\right) & =2\int_{0}^{a^{⋆}}\tau \left(a\right)p\left(a,t\right)da, \end{array}$$ (5)

for *t* > 0, where the number 2 appears because of the two newborn cells, which begin in the proliferating phase with age 0. The function, which defines the number of cells switching from quiescent to proliferating phase, *γ*(*N*), takes the form of monotone decreasing Hill function of *N*:

$$\begin{array}{c} \gamma \left(N\right)=\frac{\nu \theta^{\kappa }}{\theta^{\kappa }+N^{\kappa }}, \end{array}$$ (6)

where *ν* defines the maximal rate of cell transitioning from quiescent to proliferating population (e.g., when there are no cells, i.e., *N* = 0), *κ* is the Hill coefficient and *θ* characterises the entire cell population reaching the half maximum of *ν*. It means that the percentage of quiescent cells which enter the proliferative phase again declines to zero when the cell population rises, thus depicting density inhibition. The usage of the Hill function is motivated here to describe nonlinear and saturable mechanisms between the total cell population and the transition rate, see [36]. The number of cells that complete the division at some age in the proliferation phase are represented by function *τ*(*a*). The age *a* regulates the function *τ*(*a*) in such a way that it is almost zero until a minimum age of cells, and then it increases until the age a*:

$$\begin{array}{c} \tau \left(a\right)=\frac{\rho_{1}a^{\gamma_{1}}}{\rho_{2}^{\gamma_{1}}+a^{\gamma_{1}}}, \end{array}$$ (7)

where *ρ*_1_ is the maximum proliferation rate,*ρ*_2_ is the age at which the half-maximum effect is achieved, and the exponent *γ*_1_ is the Hill coefficient. Next we define the rate at which the cells leave the proliferating phase and become quiescent is given by the relation in (8). It depends on both age *a* and the amount of Cyclin D-CDK4/6 complex *x*_1_:

$$\begin{array}{c} \chi \left(a,x_{1}\right)=\sigma_{1}\frac{\sigma_{2}^{\gamma_{2}}}{\left(\sigma_{2}^{\gamma_{2}}+x_{1}^{\gamma_{2}}\right)}\frac{\sigma_{3}^{\gamma_{3}}}{\left(\sigma_{3}^{\gamma_{3}}+a^{\gamma_{3}}\right)}. \end{array}$$ (8)

The function *χ*(*a*, *x*_1_) determines the number of cells that do not divide because of growth-inhibiting factors. Age dependence in *χ* is motivated because the cells transit from the proliferating to quiescent phase only until a certain age that specifies a restriction point R in the cell-cycle (*G*_1_−*S* phase transition). However, until the restriction point, the concentration of Cyclin complex *x*_1_ must be under a certain threshold to allow cells to leave the proliferating phase. In Eq (8), *γ*_2_ and *γ*_3_ are Hill coefficient, *σ*_2_ and *σ*_3_ represent the concentration of Cyclin D-CDK4/6 complex *x*_1_ and age *a*, respectively, and after *γ*_2_ and *γ*_3_, the rate function *χ* asymptotically decreases to zero and thus avoiding transition of cells to quiescent phase. It indicates that at age *σ*_3_, cells are inevitably devoted to entering the proliferation compartment. Lastly, *σ*_2_ is the limit for the concentration of Cyclin’ complex, which determines R, the restriction point.

In the process of cell-signaling, cell growth is regulated by the proteins called cytokine and other proliferation governing factors, [37]. Cytokines proteins attach to their special receptors, thus activating signal transduction pathways, [38]. As per different studies, it is evident that the number of cells can be kept in balance by cytokine signals, which depend on the total cell population [39]. For detailed explanation concerning the dynamics of cytokine signals, please see [40, 41]. After quasi-steady-state approximation, the number of growth factors *g*_*f*_ stemming from the total cell number *N* is given as,

$$\begin{array}{c} g_{f}=\frac{1}{1+k_{t}N}, \end{array}$$ (9)

indicating maximum intensity, i.e., *g*_*f*_ = 1, for small cell density and effectively zero intensity for large cell densities.

### 2.2 Cell cycle model

As previously stated, we consider only four microscale states (proteins) in the cell-cycle model, which are plausible enough to incorporate reversible transition between quiescent and proliferating phase. We utilise the kinetics of Michaelis-Menten to describe the chemical reactions with enzymes and substrates from the cell-cycle, which are briefly described in the sequel. Cyclin D protein makes a complex with its catalytic partner CDK4-6 when there are sufficient growth factors. After the formation of Cyclin D-CDK4/6 complex, it phosphorylates other proteins from the cell-cycle, which are critical to advancement in the first grwoth phase of the cell-cycle and crossing the restriction point R, [29, 42]. More precisely, the Cyclin D-CDK4/6 complex phosphorylates the retinoblastoma protein Rb to inactivate it and thus release the transcription factor E2F, which in result activates many growth promoting signals to progress the cell-cycle. p21, which inhibits CDK, regulates the cell-cycle by hindering the functions of the several CDK proteins. The description of proteins is given in the Table 1. We consider the evolution of cell-cycle proteins in a single-cell whose dynamics is representative of the behavior of all cells in a population. We consider that all cells behave identical and thus one ode model with similar parameters for all cells in a population represents the microscale of underlying cell-cycle dynamics. We further postulate that our representative cell in the microscale completes division at some age *a*^⋆^, while, of course, our model accounts for the cells with shorter cycles at the macroscale via function *τ*(*a*). The following ODE system describes the cell-cycle dynamics, [43]:

$$\begin{array}{cc} \frac{dx_{1}}{da} & =k_{1s}(\frac{g_{f}}{k_{gf}+g_{f}})-k_{14}x_{4}x_{1}-k_{1d}(\frac{x_{1}}{k_{1}+x_{1}}), \end{array}$$ (10a)

$$\begin{array}{cc} \frac{dx_{2}}{da} & =k_{21}(\frac{x_{2t}-x_{2}}{k_{2}+\left(x_{2t}-x_{2}\right)})x_{1}-k_{32}x_{2}x_{3}-k_{2d}x_{2}, \end{array}$$ (10b)

$$\begin{array}{cc} \frac{dx_{3}}{da} & =k_{3s}-k_{32}x_{2}x_{3}-k_{31}(\frac{x_{3}}{k_{3}+x_{3}})x_{1}-k_{3d}x_{3}, \end{array}$$ (10c)

$$\begin{array}{cc} \frac{dx_{4}}{da} & =k_{4s}+k_{42}(\frac{k_{34}}{k_{34}+x_{3}})x_{2}-k_{41}(\frac{x_{4}}{k_{4}+x_{4}})x_{1}-k_{4d}x_{4}. \end{array}$$ (10d)

**Fig 2:**
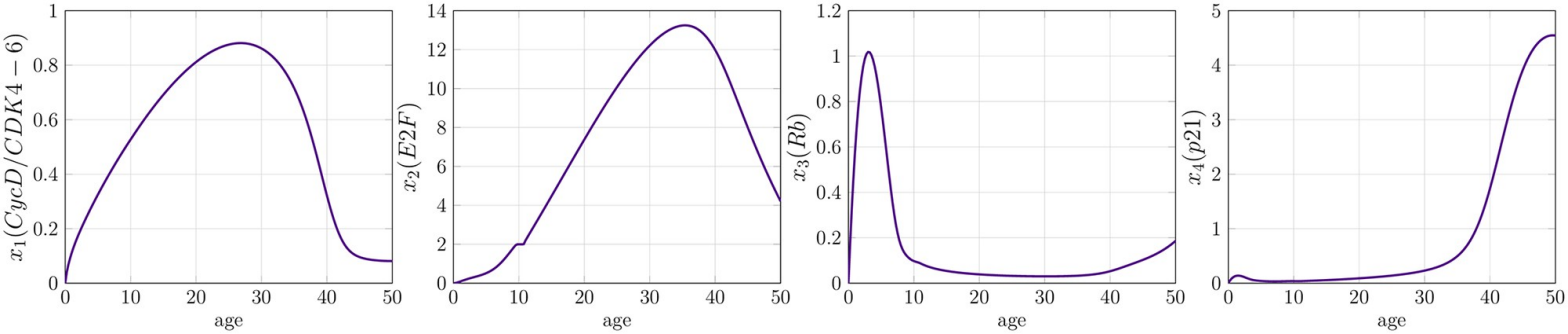
Evolution of microscale proteins from the cell-cycle. Cyclin D-CDK4/6 shows a complete activation and degradation within a full cycle. The concentration of transcription factor E2F is elevated since Retinoblastoma protein Rb is inactivated with the rise in Cyclin D-CDK4/6 complex. Similarly, protein p21 elevates near the end of the cell-cycle to help in the degradation of the Cyclin’ complex.

In Eq (10a), the first term on the right-hand side describes the synthesis of Cyclin D/CDK 4-6 complex induced by the growth factors *g*_*f*_. The last two terms describe the binding of Cyclin D/CDK 4-6 complex with tumor suppressor protein p21 and the degradation rate of Cyclin D/CDK 4-6 complex, which is induced by other cell cycle proteins, for example, Cyclin E, respectively. In Eq (10b), the first term on the right-hand side describes the synthesis of transcription factors E2F induced by Cyclin D/CDK 4-6 complex. The second term denotes the decrement of E2F due to inhibition by retinoblastoma protein Rb, while the last term depicts a constant inactivation rate of E2F induced by other cell cycle proteins, for instance, Cyclin A. In the third equation (10c), the first term on the right-hand side represents the synthesis of free un-phosphorylated retinoblastoma protein Rb. The second term denotes the decline in Rb by making a complex with E2F to inhibit it. The third term refers to the deactivation of Rb by phosphorylation from Cyclin D/CDK 4-6 complex and the last one to the degradation of Rb. In Eq (10d), the first and second terms represent the synthesis of p21 by ATM/ATR, TGF*β* pathways and induced by E2F, respectively. The third term represents the decrement in p21 due to inhibition of Cyclin D/CDK 4-6 complex, and the last term stands for the degradation of p21. The description of the parameters involved in the cell cycle model (10a)–(10d) is described below in the Table 2. The detailed derivation of the microscale model equations is not given here; however, we suggest the interested readers to read [43] for more details. For understanding, the model simulations of above mentioned four microscale states are shown in Fig 2.

**Table 1:** Description of the cell states at the microscale.

Description	State
Cyclin D-CDK4/6	*x*_1_
E2F	*x*_2_
Rb	*x*_3_
p21	*x*_4_

**Table 2:** Parameters of cell-cycle model, [25]. Here *μM* and *h* represents micromolar and hour, respectively.

Parameter	Description	Value	Unit
*k*_1*s*_	Rate constant for synthesis of Cyclin D-CDK4/6 induced by *g*_*f*_	0.155	*h* ^−1^
*k*_*gf*_	Michaelis constant for synthesis of the Cyclin D-CDK4/6 induced by *g*_*f*_	0.1	*μM*
*k*_14_	Bimolecular rate constant for binding of Cyclin D-CDK4/6 to p21	0.15	*μM* ^−1^ *h* ^−1^
*k*_1*d*_	Maximum degradation rate of Cyclin D-CDK4/6 complex	0.255	*μMh* ^−1^
*k*_1_	Michaelis constant for the degradation of Cyclin D-CDK4/6 complex	0.1	*μM*
*k*_21_	Rate constant for activation of E2F by Cyclin D-CDK4/6 complex	0.805	*h* ^−1^
*k*_2_	Michaelis constant for E2F activation by Cyclin D-CDK4/6 complex	0.01	*μM*
*x*_2*t*_	Total concentration of the transcription factor E2F	2	*μM*
*k*_32_	Bimolecular rate constant for binding of Rb to E2F	0.01	*μM* ^−1^ *h* ^−1^
*k*_2*d*_	Apparent first-order rate constant for non-specific E2F degradation	0.02	*h* ^−1^
*k*_3*s*_	Basal rate of synthesis of Rb	0.8	*h* ^−1^
*k*_31_	Rate constant for phosphorylation of Rb by Cyclin D-CDK4/6 complex	2.2	*h* ^−1^
*k*_3_	Michaelis constant for Rb phosphorylation by Cyclin D-CDK4/6 complex	0.1	*μM*
*k*_3*d*_	Apparent first-order rate constant for Rb degradation	0.01	*h* ^−1^
*k*_4*s*_	Basal, E2F-independent rate of synthesis of p21	0.8	*μMh* ^−1^
*k*_42_	Rate constant for synthesis of p21 induced by E2F	0.1	*h* ^−1^
*k*_34_	Constant of inhibition by Rb of p21 synthesis	0.1	*μM*
*k*_41_	Rate constant for p21 inactivation via phosphorylation by Cyclin D-CDK4/6 complex	50	*h* ^−1^
*k*_4_	Michaelis constant for p21 phosphorylation by Cyclin D-CDK4/6	0.5	*μM*
*k*_4*d*_	Apparent first-order rate constant for non-specific p21 degradation	0.06	*h* ^−1^

## 3 Existence and uniqueness of non-negative solution

This section shows that the initial-boundary value problem (1)–(5), (10a)–(10d) has a unique solution. For simplicity, we will use the cell-cycle model for the whole time *t* and not just with respect to age *a*. First, we define the Banach spaces, *X* = *L*^1^(0, *a*^⋆^) × *L*^1^(0, *a*^⋆^) and *Y* = *L*^1^(0, *a*^⋆^) × *L*^1^(0, *a*^⋆^) × *L*^1^(0, *a*^⋆^) × *L*^1^(0, *a*^⋆^) with the norm $\left\|\phi \right\|=\sum_{i=1}^{2}{\left\|\phi_{i}\right\|}_{1}$ for *ϕ*(*a*) = (*ϕ*_1_(*a*), *ϕ*_2_(*a*))^T^ ∈ *X* and $\left\|\varphi \right\|=\sum_{i=1}^{4}{\left\|\varphi_{i}\right\|}_{1}$ for *φ*(*a*) = (*φ*_1_(*a*), *φ*_2_(*a*), *φ*_3_(*a*), *φ*_4_(*a*))^T^ ∈ *Y*, where ‖ ⋅ ‖_1_ is ordinary norm of *L*^1^(0, *a*^⋆^). First, we take the initial-boundary value problem of the system (1)–(5) as an abstract Cauchy problem on the Banach space *X*. Further, assume that $g_{a},g_{aa}\in L^{\infty }\left(\left(0,a^{⋆}\right)\times R^{+}\right)$, and death rates are non-negative, i.e., *μ*_*p*_(⋅) = *μ*_*q*_(⋅)≥0, locally integrable on [0, *a*^⋆^). The transition rate *χ*(*a*, *x*_1_) ∈ *L*^∞^((0, *a*^⋆^) × (0, *a*^⋆^)), and *τ*(*a*) ∈ *L*^1^(0, *a*^⋆^). Now, first we define a linear operator $A_{1}$:

$$\begin{array}{c} \left(A_{1}\phi \right)\left(a\right)=(\begin{array}{c} -\mu_{q}\left(a\right)\phi_{1}\left(a\right)\\ -\frac{\partial (g\left(a\right)\phi_{2}\left(a\right))}{\partial a}-\left(\tau \left(a\right)+\mu_{p}\left(a\right)\right)\phi_{2}\left(a\right) \end{array}),\;\;\phi \left(a\right)={\left(\phi_{1}\left(a\right),\phi_{2}\left(a\right)\right)}^{T}\in D\left(A_{1}\right), \end{array}$$

where T depicts the vector’s transpose and the domain $D(A_{1})$ is defined below

$$D\left(A_{1}\right)=\{\left(\phi_{1},\phi_{2}\right)\;|\;\phi_{i}\;\;\text{is}\;\text{absolute}\;\text{continuous}\;\text{on}\;\left[0,a^{⋆}\right),\phi \left(0\right)={\left(0,2\int_{0}^{a^{⋆}}\tau \left(a\right)\phi_{2}\left(a\right)da\right)}^{T}\}.$$

The nonlinear operator $F_{1}:X\times Y\to X$ is given by

$$\begin{array}{c} \left(F_{1}\left(\phi ,\varphi \right)\right)\left(a\right)=(\begin{array}{c} \frac{-\nu \theta^{\kappa }\phi_{1}\left(a\right)}{\theta^{\kappa }+{\left(N\phi \right)}^{\kappa }}+\chi \left(\varphi_{1},a\right)\phi_{2}\left(a\right)\\ \frac{\nu \theta^{\kappa }\phi_{1}\left(a\right)}{\theta^{\kappa }+{\left(N\phi \right)}^{\kappa }}-\chi \left(\varphi_{1},a\right)\phi_{2}\left(a\right) \end{array}),\;\phi \in X,\;\;\varphi \in Y, \end{array}$$

where the linear operator *N* on *L*^1^(0, *a*^⋆^) × *L*^1^(0, *a*^⋆^) is given by

$$\begin{array}{c} N\phi =\int_{0}^{a^{⋆}}(\phi_{1}\left(a\right)+\phi_{2}\left(a\right))da. \end{array}$$

Let *υ*(*t*) = (*q*(⋅, *t*), *p*(⋅, *t*))^T^ ∈ *X*. We can define an initial-boundary value problem (1)–(5) in the form of an abstract semilinear IVP in *X*:

$$\begin{array}{c} \frac{d}{dt}\upsilon \left(t\right)=A_{1}\upsilon \left(t\right)+F_{1}\left(\upsilon \left(t\right),v\left(t\right)\right),\;\upsilon \left(0\right)=\upsilon_{0}\in X, \end{array}$$ (11)

where *υ*_0_(*a*) = (*q*_0_(*a*), *p*_0_(*a*)).

Next, we define initial value problem (10a)–(10d) as Cauchy problem on the Banach space *Y*. Let $A_{2}$ be a linear operator written as follows

$$\begin{array}{c} \left(A_{2}\varphi \right)\left(a\right)=(\begin{array}{c} 0\\ -k_{2d}\varphi_{2}\left(a\right)\\ k_{3s}-k_{3d}\varphi_{3}\left(a\right)\\ k_{4s}-k_{4d}\varphi_{4}\left(a\right) \end{array}),\;\;\;\varphi \left(a\right)={\left(\varphi_{1}\left(a\right),\varphi_{2}\left(a\right),\varphi_{3}\left(a\right),\varphi_{4}\left(a\right)\right)}^{T}\in D\left(A_{2}\right), \end{array}$$

where the domain $D(A_{2})$ is

$$D\left(A_{2}\right)=\left\{\varphi \in Y\;|\;\varphi_{i}\;\;\text{is}\;\text{absolute}\;\text{continuous}\;\text{on}\;\left[0,a^{⋆}\right),\;\;\varphi \left(0\right)={\left(0,0,0,0\right)}^{T}\right\}.$$

We define the nonlinear operator $F_{2}:X\times Y\to Y$ by

$$\left(F_{2}\left(\varphi ,\varphi \right)\right)\left(a\right)=(\begin{array}{c} k_{1s}(\frac{g_{f}\left(N\varphi \right)}{k_{gf}+g_{f}\left(N\varphi \right)})-k_{14}\varphi_{4}\left(a\right)\varphi_{1}\left(a\right)-k_{1d}(\frac{\varphi_{1}\left(a\right)}{k_{1}+\varphi_{1}\left(a\right)}),\\ k_{21}(\frac{x_{2t}-\varphi_{2}\left(a\right)}{k_{2}+\left(x_{2t}-\varphi_{2}\left(a\right)\right)})\varphi_{1}\left(a\right)-k_{32}\varphi_{2}\left(a\right)\varphi_{3}\left(a\right)\\ -k_{32}\varphi_{2}\left(a\right)\varphi_{3}\left(a\right)-k_{31}(\frac{\varphi_{3}\left(a\right)}{k_{3}+\varphi_{3}\left(a\right)})\varphi_{1}\left(a\right)\\ k_{42}(\frac{k_{34}}{k_{34}+\varphi_{3}\left(a\right)})\varphi_{2}\left(a\right)-k_{41}(\frac{\varphi_{4}\left(a\right)}{k_{4}+\varphi_{4}\left(a\right)})\varphi_{1}\left(a\right) \end{array}),$$

where *ϕ* ∈ *X*, *φ* ∈ *Y*. Let $v\left(t\right)={\left(x_{1}\left(t\right),x_{2}\left(t\right),x_{3}\left(t\right),x_{4}\left(t\right)\right)}^{T}\in Y$. Then the initial-boundary value problem (10a)–(10d) can be defined as an abstract semilinear IVP in *Y*:

$$\begin{array}{c} \frac{d}{dt}v\left(t\right)=A_{2}v\left(t\right)+F_{2}\left(\upsilon \left(t\right),v\left(t\right)\right),\;v\left(0\right)=v_{0}\in Y, \end{array}$$ (12)

where $v_{0}\left(t\right)=\left(x_{1}^{0},x_{2}^{0},x_{3}^{0},x_{4}^{0}\right)$. Now, we can define a combine Cauchy problem for (11) and (12) as follows:

$$\begin{array}{c} \frac{d}{dt}(\begin{array}{c} \upsilon \\ v \end{array})=(\begin{array}{cc} A_{1} & 0\\ 0 & A_{2} \end{array})(\begin{array}{c} \upsilon \\ v \end{array})+(\begin{array}{c} F_{1}\left(\upsilon ,v\right)\\ F_{2}\left(\upsilon ,v\right) \end{array}),\;(\begin{array}{c} \upsilon (0)\\ v(0) \end{array})=(\begin{array}{c} \upsilon_{0}\\ v_{0} \end{array})\in Z, \end{array}$$

$$\begin{array}{c} \frac{d}{dt}\zeta \left(t\right)=A\zeta \left(t\right)+F\left(\zeta \left(t\right)\right),\;\zeta \left(0\right)=\zeta_{0}\in Z, \end{array}$$ (13)

where *ζ* = (*υ*, *v*), *ζ*_0_ = (*υ*_0_, *v*_0_), $A=(\begin{array}{cc} A_{1} & 0\\ 0 & A_{2} \end{array})$, $F=(\begin{array}{c} F_{1}\\ F_{2} \end{array})$ and *Z* = {*X*, *Y*} is a Banach space.*T*(*t*) is *C*_0_-semigroup which is generated by A, for all *t* ≥ 0 and operator F exhibits continuous Frechet differentiabilityon *Z* (in other words, $F_{1}$ and $F_{2}$ are Frechet differentiable on both *X* and *Y*, see Lemma A.1 in the appendix, where we show Frechet differentiability of $F_{1}$ from *X* → *X*). Then there exists a maximum interval [0, *t*_1_) for existence and uniqueness of continuous mild solution *t* → *ζ*(*t*, *ζ*_0_) from [0, *t*_1_) to *Z* for each *ζ*_0_ ∈ *Z*, so that

$$\begin{array}{c} \zeta \left(t,\zeta_{0}\right)=T\left(t\right)\zeta_{0}+\int_{0}^{t}T\left(t-s\right)F\left(\zeta \left(s,\zeta_{0}\right)\right)ds,\;\;\forall t\in \left[0,t_{1}\right) \end{array}$$ (14)

and *t*_1_ = + ∞ or ${\text{lim}}_{t\to t_{1}^{-}}\left\|\zeta \left(t,\zeta_{0}\right)\right\|=\infty$. Additionally, when $\zeta_{0}\in D\left(A\right)$, then $\zeta \left(t,\zeta_{0}\right)\in D\left(A\right)$ for 0 ≤ *t* < *t*_1_ and the function *ζ* → *ζ*(*t*, *ζ*_0_) is continuously differentiable which also satisfies (13) on [0, *t*_1_), see Proposition 4.16 [44] and [45].

**Remark 3.1**. *We denote the maximum value of the solution variables as* *p*_max_, *q*_max_, *x*_1,max_, *x*_2,max_, *x*_3,max_ *and* *x*_4,max_. *If we normalise the governing equations using* $N\left(a\right)=p\left(a,t\right)+q\left(a,t\right)+x_{1}\left(a\right)+x_{2}\left(a\right)+x_{3}\left(a\right)+x_{4}\left(a\right)$, *then an a-priori estimate on these would lead to* $p\left(a,t\right)+q\left(a,t\right)+x_{1}\left(a\right)+x_{2}\left(a\right)+x_{3}\left(a\right)+x_{4}\left(a\right)=1$.

**Lemma 3.2**. *Let* $\Omega =\{\left(p,q,x_{1},x_{2},x_{3},x_{4}\right)\in Z|p\geq 0,Q\geq 0,x_{1}\geq 0,x_{2}\geq 0,x_{3}\geq 0,x_{4}\geq 0\}$ *and let* $\Omega_{0}=\left\{\left(p,q,x_{1},x_{2},x_{3},x_{4}\right)\in Z|0\leq p\leq p_{\text{max}},0\leq q\leq q_{\text{max}},0\leq x_{1}\leq x_{1,\text{max}},0\leq x_{2}\leq x_{2,\text{max}},0\leq x_{3}\leq x_{3,\text{max}},0\leq x_{4}\leq x_{4,\text{max}}\right\}$. *Then, the mild solution* *ζ*(*t*, *ζ*_0_), *ζ*_0_ ∈ Ω *of* (13), *after a finite time, enters* Ω_0_ *which is positively invariant*.

*Proof*. First, we derive the solution expression from (1) as follows:

$$\begin{array}{cc} q(a,t):= & \text{exp}(\;-\;\int_{0}^{t}\mu_{q}\left(a\right)+\gamma \left(N\left(t\right)\right)dt)\{\;\int_{0}^{t}\text{exp}(\;-\;\int_{0}^{\xi }\mu_{q}\left(a\right)+\gamma \left(N\left(\pi \right)\right)d\pi )\\ & \chi \left(x_{1}\left(a\right),a\right)p\left(a,\xi \right)d\xi +q_{0}\left(a\right)\}. \end{array}$$ (15)

and, immediately, it follows that *q*(*a*, *t*) ≥ 0 when *q*_0_(*a*) ≥ 0 and *p*(*a*, *t*) is positive. Next, to derive the solution of Eq (2), we first use transformations $\tilde{p}\left(a,t\right)=g\left(a\right)p\left(a,t\right)$ and $\tilde{q}\left(a,t\right)=g\left(a\right)q\left(a,t\right)$ for *t* ∈ [0, *t*_1_] and *a* ∈ [*a*_0_, *a*^⋆^). Then for all *t* ∈ (0, *t*_1_) and *a* ∈ (*a*_0_, *a*^⋆^), we have from Eq (2):

$$\begin{array}{c} \frac{\partial \tilde{p}\left(a,t\right)}{\partial t}+g\left(a\right)\frac{\partial \tilde{p}\left(a,t\right)}{\partial a}=\gamma \left(N\left(t\right)\right)\tilde{q}\left(a,t\right)-\left(\tau \left(a\right)+\chi \left(x_{1}\left(a\right),a\right)+\mu_{p}\left(a\right)\right)\tilde{p}\left(a,t\right),\; \end{array}$$ (16)

Following that, we utilize the parameter transform given in Lemma 3.1 [40] in order to eliminate the term *g*(*a*) and introduce *η* as a new age variable for both *p* and *q*. We obtain

$$\frac{\partial }{\partial \eta }\tilde{p}\left(a\left(\eta \right),t\right)=\frac{da}{d\eta }\frac{\partial }{\partial a}\tilde{p}\left(a,t\right)=g\left(a\right)\frac{\partial }{\partial a}\tilde{p}\left(a,t\right),\;\;\text{where}\;\frac{da}{d\eta }=g\left(a\right).$$

Therefore, from Eq (16), it follows that

$$\begin{array}{cc} \;\frac{\partial \tilde{p}\left(a\left(\eta \right),t\right)}{\partial t}+\frac{\partial \tilde{p}\left(a\left(\eta \right),t\right)}{\partial \eta }= & \gamma \left(N\left(t\right)\right)\tilde{q}\left(a\left(\eta \right),t\right)-(\tau \left(a\left(\eta \right)\right)+\chi \left(x_{1}\left(a\left(\eta \right)\right),a\left(\eta \right)\right)\\ & +\mu_{p}\left(a\left(\eta \right)\right))\tilde{p}\left(a\left(\eta \right),t\right).\; \end{array}$$ (17)

To determine the explicit relation of $\tilde{p}\left(a\left(\eta \right),t\right)$, employ the method of characteristics (MOC). We suppose that $\tilde{p}\left(a\left(\eta \right),t\right)$ is characterized by an ordinary differential equation along the curve $\left(a\left(\psi_{1}\left(y\right)\right),\psi_{2}\left(y\right)\right)=\psi \left(y\right)$, then

$$\begin{array}{c} {\dot{\psi }}_{1}\left(y\right)≔1\Rightarrow \psi_{1}\left(y\right)=y+c_{1},\;\;{\dot{\psi }}_{2}\left(y\right)≔1\Rightarrow \psi_{2}\left(y\right)=y+c_{2},\;\;z\left(y\right)≔\tilde{p}\left(a\left(\psi_{1}\left(y\right)\right),\psi_{2}\left(y\right)\right), \end{array}$$

where $c_{1},c_{2}\in R$ are constants. Then, it follows

$$\begin{array}{cc} \frac{dz}{dy}= & \frac{d\tilde{p}\left(a\left(\psi_{1}\left(y\right)\right),\psi_{2}\left(y\right)\right)}{dy}\\ = & \frac{\partial \tilde{p}\left(a\left(\psi_{1}\left(y\right)\right),\psi_{2}\left(y\right)\right)}{\partial a}\;\frac{da(\psi_{1}\left(y\right))}{d\psi_{1}}\;\frac{d\psi_{1}\left(y\right)}{dy}+\frac{\partial \tilde{p}\left(a\left(\psi_{1}\left(y\right)\right),\psi_{2}\left(y\right)\right)}{\partial \psi_{2}}\;\frac{d\psi_{2}\left(y\right)}{dy}\\ = & \gamma \left(N\left(\psi_{2}\left(y\right)\right)\right)\tilde{q}\left(a\left(\psi_{1}\left(y\right)\right),\psi_{2}\left(y\right)\right)-(\tau \left(a\left(\psi_{1}\left(y\right)\right)\right)+\chi \left(x_{1}\left(a\left(\psi_{1}\left(y\right)\right)\right),a\left(\psi_{1}\left(y\right)\right)\right)\\ & +\mu_{p}\left(a\left(\psi_{1}\left(y\right)\right)\right))\tilde{p}\left(a\left(\psi_{1}\left(y\right)\right),\psi_{2}\left(y\right)\right)\\ = & \gamma \left(N\left(\psi_{2}\left(y\right)\right)\right)\tilde{q}\left(a\left(\psi_{1}\left(y\right)\right),\psi_{2}\left(y\right)\right)-(\tau \left(a\left(\psi_{1}\left(y\right)\right)\right)+\chi \left(x_{1}\left(a\left(\psi_{1}\left(y\right)\right)\right),a\left(\psi_{1}\left(y\right)\right)\right)\\ & +\mu_{p}\left(a\left(\psi_{1}\left(y\right)\right)\right))z\left(y\right). \end{array}$$ (18)

We can now write $\tilde{p}$ using an ODE (18) so that

$$\begin{array}{c} \tilde{p}(a(y+c_{1}),y+c_{2})\;=\tilde{p}\left(a\left(\psi_{1}\left(y\right)\right),\psi_{2}\left(y\right)\right)=z\left(y\right)\\ \;=\text{exp}(-\int_{0}^{y}(\tau \left(a\left(\psi_{1}\left(\xi \right)\right)\right)+\chi \left(x_{1}\left(a\left(\psi_{1}\left(\xi \right)\right)\right),a\left(\psi_{1}\left(\xi \right)\right)\right)+\mu_{p}\left(a\left(\psi_{1}\left(\xi \right)\right)\right))d\xi )\\ \;[\int_{0}^{y}\text{exp}(\int_{0}^{\zeta }(\tau \left(a\left(\psi_{1}\left(\xi \right)\right)\right)+\chi \left(x_{1}\left(a\left(\psi_{1}\left(\xi \right)\right)\right),a\left(\psi_{1}\left(\xi \right)\right)\right)+\mu_{p}\left(a\left(\psi_{1}\left(\xi \right)\right)\right))d\xi )\\ \;\gamma \left(N\left(\psi_{2}\left(\zeta \right)\right)\right)\tilde{q}\left(a\left(\psi_{1}\left(\zeta \right)\right),\psi_{2}\left(\zeta \right)\right)d\zeta +\tilde{p}\left(a\left(\psi_{1}\left(0\right)\right),\psi_{2}\left(0\right)\right)]\\ \;=\text{exp}(-\int_{0}^{y}(\tau \left(a\left(\xi +c_{1}\right)\right)+\chi \left(x_{1}\left(a\left(\psi_{1}\left(\xi +c_{1}\right)\right)\right),a\left(\xi +c_{1}\right)\right)+\mu_{p}\left(a\left(\xi +c_{1}\right)\right))d\xi )\\ \;[\int_{0}^{y}\;\text{exp}(\;\int_{0}^{\zeta }\;(\tau \left(a\left(\xi +c_{1}\right)\right)\;+\chi \left(x_{1}\left(a\left(\psi_{1}\left(\xi +c_{1}\right)\right)\right),a\left(\xi +c_{1}\right)\right)\;+\;\mu_{p}\left(a\left(\xi +c_{1}\right)\right))d\xi )\\ \;\gamma \left(N\left(\zeta +c_{2}\right)\right)\tilde{q}\left(a\left(\zeta +c_{1}\right),\zeta +c_{2}\right)d\zeta +\tilde{p}\left(a\left(c_{1}\right),c_{2}\right)]. \end{array}$$

Following that, we establish the boundary set Γ ≔ {[*a*_0_, *a*^⋆^) × {0}} ∪ {{0} × [0, *t*_1_]} in such a way that if a curve $\left(a\left(\psi_{1}\left(y\right)\right),\psi_{2}\left(y\right)\right)$ begins in Γ, we may utilize the boundary condition to determine $\tilde{p}\left(a\left(c_{1}\right),c_{2}\right)$. If we want (*a*(*y*+ *c*_1_), *y* + *c*_2_) to be in Γ, then either *c*_1_ = 0 or *c*_2_ = 0. This results in the two situations below. In the first scenario, *c*_1_ = 0 and *c*_2_ ∈ [0, *t*_1_) can be chosen randomly. Then, in this case,

$$\begin{array}{cc} & \tilde{p}(a\left(y\right),y+c_{2})=\text{exp}(-\int_{0}^{y}(\tau \left(a\left(\xi \right)\right)+\chi \left(x_{1}\left(a\left(\xi \right)\right),a\left(\xi \right)\right)+\mu_{p}\left(a\left(\xi \right)\right))d\xi )\\ & \;[\int_{0}^{y}\text{exp}(\int_{0}^{\zeta }(\tau \left(a\left(\xi \right)\right)+\chi \left(x_{1}\left(a\left(\xi \right)\right),a\left(\xi \right)\right)+\mu_{p}\left(a\left(\xi \right)\right))d\xi )\gamma \left(N\left(\zeta +c_{2}\right)\right)\\ & \;\tilde{q}\left(a\left(\zeta \right),\zeta +c_{2}\right)d\zeta +\tilde{p}\left(a\left(0\right),c_{2}\right)]. \end{array}$$

We may now utilize the characteristic solution to achieve the solution in {(*a*(*η*), *t*)|*t* ∈ [0, *t*_1_], *η* ∈ [0, min(η*, *t*))}:

$$\eta \overset{!}{=}\psi_{1}\left(y\right)=y+c_{1}=y\Rightarrow y=\eta \;\;\text{and}\;\;t\overset{!}{=}\psi_{2}\left(y\right)=y+c_{2}\Rightarrow c_{2}=t-y,$$

which implies

$$\begin{array}{cc} \tilde{p}\left(a\left(\eta \right),t\right)= & \text{exp}(-\int_{0}^{\eta }(\tau \left(a\left(\xi \right)\right)+\chi \left(x_{1}\left(a\left(\xi \right)\right),a\left(\xi \right)\right)+\mu_{p}\left(a\left(\xi \right)\right))d\xi )\\ & [\int_{0}^{\eta }\text{exp}(\int_{0}^{\zeta }(\tau \left(a\left(\xi \right)\right)+\chi \left(x_{1}\left(a\left(\xi \right)\right),a\left(\xi \right)\right)+\mu_{p}\left(a\left(\xi \right)\right))d\xi )\gamma \left(N\left(\zeta +t-\eta \right)\right)\\ & \tilde{q}\left(a\left(\zeta \right),\zeta +t-\eta \right)d\zeta +\tilde{p}\left(a\left(0\right),t-\eta \right)]. \end{array}$$

This establishes the equation for *g*(*a*(*η*))*p*(*a*(*η*), *t*) in case of *η* < *t*. Then, we take *c*_1_ ∈ [0, η*) is arbitrary and *c*_2_ = 0. Then we achieve,

$$\begin{array}{cc} \tilde{p}\left(a\left(y+c_{1}\right),u\right)= & \text{exp}(\;-\;\int_{0}^{y}(\tau \left(a\left(\xi +c_{1}\right)\right)+\chi \left(x_{1}\left(a\left(\xi +c_{1}\right)\right),a\left(\xi +c_{1}\right)\right)+\mu_{p}\left(a\left(\xi +c_{1}\right)\right))\\ & d\xi )[\int_{0}^{y}\text{exp}(\int_{0}^{\zeta }(\tau \left(a\left(\xi +c_{1}\right)\right)+\chi \left(x_{1}\left(a\left(\xi +c_{1}\right)\right),a\left(\xi +c_{1}\right)\right)\\ & +\mu_{p}\left(a\left(\xi +c_{1}\right)\right))d\xi )+\gamma \left(N\left(\zeta \right)\right)\tilde{q}\left(a\left(\zeta +c_{1}\right),\zeta \right)d\zeta +\tilde{p}\left(a\left(c_{1}\right),0\right)]. \end{array}$$

We may now utilize the characteristic solution to achieve a solution in {(*a*(*η*), *t*)|*t* ∈ [0, *t*_1_], *η* ∈ [*t*, η*)}:

$$\eta \overset{!}{=}\psi_{1}\left(y\right)=y+c_{1}\Rightarrow c_{1}=\eta -y\;\;\text{and}\;\;t\overset{!}{=}\psi_{2}\left(y\right)=y+c_{2}\Rightarrow y=t,$$

which results into

$$\begin{array}{cc} \tilde{p}\left(a\left(\eta \right),t\right)= & \text{exp}(-\int_{0}^{t}(\tau \left(a\left(\xi +\eta -t\right)\right)+\chi \left(x_{1}\left(a\left(\xi +\eta -t\right)\right),a\left(\xi +\eta -t\right)\right)\\ & +\mu_{p}\left(a\left(\xi +\eta -t\right)\right))d\xi )[\int_{0}^{t}\text{exp}(\int_{0}^{\zeta }(\tau \left(a\left(\xi +\eta -t\right)\right)\\ & +\chi \left(x_{1}\left(a\left(\xi +\eta -t\right)\right),a\left(\xi +\eta -t\right)\right)+\mu_{p}\left(a\left(\xi +\eta -t\right)\right))d\xi )\\ & \gamma \left(N\left(\zeta \right)\right)\tilde{q}\left(a\left(\zeta +\eta -t\right),\zeta \right)d\zeta +\tilde{p}\left(a\left(\eta -t\right),0\right)]. \end{array}$$

This establishes the equation for *g*(*a*(*η*))*p*(*a*(*η*), *t*) for *η* > *t*. Thus, the final solution for *g*(*a*(*η*))*p*(*a*(*η*), *t*) can be written as:

$$\begin{array}{c} \tilde{p}\left(a\left(\eta \right),t\right)≔\{\begin{array}{cc} & \text{exp}(-\int_{0}^{\eta }(\tau \left(a\left(\xi \right)\right)+\chi \left(x_{1}\left(a\left(\xi \right)\right),a\left(\xi \right)\right)+\mu_{p}\left(a\left(\xi \right)\right))d\xi )[h\left(t-\eta \right)\\ & \int_{0}^{\eta }\;\text{exp}(\;\int_{0}^{\zeta }\;(\tau \left(a\left(\xi \right)\right)\;+\;\chi \left(x_{1}\left(a\left(\xi \right)\right),a\left(\xi \right)\right)\;+\;\mu_{p}\left(a\left(\xi \right)\right))d\xi )\\ & \gamma \left(N\left(\zeta +t-\eta \right)\right)\tilde{q}\left(a\left(\zeta \right),\zeta +t-\eta \right)d\zeta ],\;\bar{a}<t\\ & \text{exp}(-\int_{0}^{t}(\tau \left(a\left(\xi +\eta -t\right)\right)+\chi \left(x_{1}\left(a\left(\xi +\eta -t\right)\right),a\left(\xi +\eta -t\right)\right)+\\ & \mu_{p}\left(a\left(\xi +\eta -t\right)\right))d\xi )[p_{0}\left(a\left(\eta -t\right)\right)+\int_{0}^{t}\text{exp}(\int_{0}^{\zeta }(\tau \left(a\left(\xi +\eta -t\right)\right)\\ & +\chi \left(x_{1}\left(a\left(\xi +\eta -t\right)\right),a\left(\xi +\eta -t\right)\right)+\mu_{p}\left(a\left(\xi +\eta -t\right)\right))d\xi )\gamma \left(N\left(\zeta \right)\right)\\ & \tilde{q}\left(a\left(\zeta +\eta -t\right),\zeta \right)d\zeta ],\;\bar{a}\geq t. \end{array} \end{array}$$

where, *h*(*t* − *η*) denotes the boundary condition $\tilde{p}\left(a\left(0\right),t-\eta \right)$. It can be seen that above relation for *g*(*a*)*p*(*a*, *t*) is positive for positive initial data and when *g*(*a*)*q*(*a*, *t*) ≥ 0.

Next, we check the positivity of coupled ODE model (10a)–(10d). Thereby, the set of ODEs are written as

$$\begin{array}{c} \left\{ \begin{array}{cc} \frac{dx_{1}}{da} & =f_{1}\left(x_{1},x_{4}\right),\\ \frac{dx_{2}}{da} & =f_{2}\left(x_{1},x_{2},x_{3}\right),\\ \frac{dx_{3}}{da} & =f_{3}\left(x_{1},x_{2},x_{3}\right),\\ \frac{dx_{4}}{da} & =f_{4}\left(x_{1},x_{2},x_{3},x_{4}\right), \end{array}\right. \end{array}$$ (19)

where *f*_1_, *f*_2_, $f_{3}$ and $f_{4}$ represent the vector fields of the corresponding microscale states *x*_1_-$x_{4}$. Note that in Eq (19), *f*_1_ does not show any dependence on *N* (or, in other words, dependence on *p* and *q*) because *N* varies with time, and at each time step, it is a fixed constant which determines growth factors for all ages. Next, in order to check the positivity of the solutions of all ODEs in this case, it is sufficient to know that the vector fields $f_{1},f_{2},f_{3},f_{4}$ are continuously differentiable and are pointing away from the negative parts in the state space. Starting with the ODE for *x*_1_ from (19), we substitute $x_{4}=0$ in $f_{1}\left(x_{1},x_{4}\right)$, which yields ${\dot{x}}_{1}=f_{1}\left(x_{1}\right)$. It can be seen that $f_{1}\left(x_{1}\right)=k_{1s}(\frac{g_{f}}{k_{gf}+g_{f}})-k_{1d}(\frac{x_{1}}{k_{1}+x_{1}})>0$ for all *a* > 0, when $k_{1s}(\frac{g_{f}}{k_{gf}+g_{f}})>k_{1d}(\frac{x_{1}}{k_{1}+x_{1}})$, which means that the concentration of *x*_1_ increases more than it decreases for all ages. It is evident since growth factors are the only source of increase in the concentration of *x*_1_. Therefore, when growth factors are at the absolute minimum, *x*_1_ is also at its lowest concentration, and hence the decrement (or degradation) cannot be more than the activation of complex *x*_1_. Since the solution to the system (10a)–(10b) is unique for each given initial condition (evident from (13) and (14)), thus for any given $x_{4}>0$, the solution will remain in the first quadrant. This guarantees the positivity of solution for *x*_1_. Next, we assume *x*_1_ = 0 in $f_{2}\left(x_{1},x_{2},x_{3}\right)$ which yields an ODE ${\dot{x}}_{2}=f_{2}\left(x_{2},x_{3}\right)$. The solution to which takes the form $x_{2}\left(a\right)=x_{2}^{0}e^{-(k_{32}x_{3}\left(a\right)-k_{2d})a}$, which implies $x_{2}\left(a\right)>0$ for all $x_{2}^{0}>0$ as well as for all values of $x_{3}\left(a\right)$. Thus for any given positive initial data, the solution $x_{2}\left(a\right)$ is positive for all ages. In the similar fashion, we can now substitute $x_{3}=0$ in $f_{2}\left(x_{1},x_{2},x_{3}\right)$ which yields a nonlinear ODE ${\dot{x}}_{2}=f_{2}\left(x_{1},x_{2}\right)$. The explicit solution cannot be computed in this case. However, the phase portrait of $\left(x_{1},x_{2}\right)$ shows that the solution trajectories point away from the axis which separate the positive and negative space for given positive initial data. In a similar way, we can also derive sufficient conditions for the positivity of the solutions for $x_{3}\left(a\right)$ and $x_{4}\left(a\right)$. With this, we attain that, if *ζ*_0_ ∈ Ω, *ζ* (*t*, *ζ*_0_) ∈ Ω∀*t* > 0.

Now, suppose *z*(*t*, ⋅) = *q*(*t*, ⋅)+ *p*(*t*, ⋅) and death rates are identical, i.e., *μ*_*p*_ = *μ*_*q*_. Then, we have from Eqs (1) and (2):

$$\begin{array}{c} \frac{dz(t,\cdot )}{dt}=Bz\left(t,\cdot \right)-(\frac{\partial }{\partial a}+\frac{\tau (a)}{g(a)})g\left(a\right)p\left(t,\cdot \right),\;z\left(0\right)=q_{0}\left(0\right)+p_{0}\left(0\right)\in L^{1}\left(0,a^{⋆}\right), \end{array}$$ (20)

where we define operator B as B = −*μ*_*p*_(*a*) and

$$D\left(B\right)=\{\phi \in L^{1}\left(0,a^{⋆}\right)|\phi \;\;\text{is}\;\text{absolute}\;\text{continuous}\;\text{on}\;\left[0,a^{⋆}\right)\;\;\text{and}\;\;\varphi \left(0\right)=0\}.$$

From Eq (20), it leads to

$$\begin{array}{c} z\left(t,\cdot \right)=W\left(t\right)z\left(0,\cdot \right)-\int_{0}^{t}U\left(t-s\right)(\frac{\partial }{\partial a}+\frac{\tau (a)}{g(a)})g\left(a\right)p\left(s,\cdot \right)ds, \end{array}$$ (21)

where operator B generates a positive *C*_0_-semigroup W(*t*). As we know, W(*t*) is a nilpotent translation semigroup, it leads to *z*(*t*)(*a*) ≤ *q*_0_(*a* − *t*) + *p*_0_(*a* − *t*), *a* > *t* and *z*(*t*) ≤ 0 for *t* ≥ *a*^⋆^. Therefore, the mild solution *ζ*(*t*, *ζ*_0_), *ζ*_0_ ∈ Ω enters Ω_0_ for *t* ≥ Ω, and in case of *ζ*_0_ ∈ Ω_0_, *ζ* (*t*, *ζ*_0_) ∈ Ω_0_, ∀*t* ≥ 0. Hence proved.

We conclude from the above result that the norm of the local solution $\zeta \left(t,\zeta_{0}\right),\;\zeta_{0}\in D\left(A\right)\cap \Omega$, of (13) is defined and hence finite. As a result, we achieve the final result.

**Theorem 3.3**. *The abstract Cauchy problem* (13) *has a unique global classical solution on* *Z* *with respect to the initial data* $z_{0}\in \Omega \cap D\left(A\right)$.

Consequently, given a positive initial data, the IVP (1) and (2) has a unique positive solution.

## 4 Existence and stability of steady-state

Here, we establish the steady-state solution of the model and sufficient conditions for the existence of the positive steady-state. First, we introduce some notations in the sequel. Let’s define *X* as a real/complex Banach space and *X*^⋆^ be its dual space. The notation 〈*F*, *ψ*〉 represents the value of *F* ∈ *X*^⋆^ at *ψ* ∈ *X*. A cone *X*_+_ is defined by the following:

$$X_{+}\neq \left\{0\right\},\;X_{+}\cap \left(-X_{+}\right)=\left\{0\right\},\;\lambda X_{+}\subset X_{+},\lambda \geq 0,\;X_{+}+X_{+}\subset X_{+}.$$

Moreover, the dual cone, represented as $X_{+}^{⋆}$, is the subset of the dual space.

### 4.1 Existence of steady-states

Let $\bar{p}$, $\bar{q}$, ${\bar{x}}_{1}-{\bar{x}}_{4}$ represent the steady-states of the system (1) and (2), (10a)–(10d). Then, $\bar{p}$, $\bar{q}$, ${\bar{x}}_{1}-{\bar{x}}_{4}$ must satisfy these time-invariant set of ODEs:

$$\begin{array}{c} \left\{ \begin{array}{cc} 0 & =\chi \left(a,{\bar{x}}_{1}\right)\bar{p}\left(a\right)-\left(\bar{\gamma }+\mu_{q}\left(a\right)\right)\bar{q}\left(a\right),\\ \partial_{a}\left(g\left(a\right)\bar{p}\left(a\right)\right) & =\bar{\gamma }\bar{q}\left(a\right)-\left(\tau \left(a\right)+\chi \left(a,{\bar{x}}_{1}\right)+\mu_{p}\left(a\right)\right)\bar{p}\left(a\right),\\ \bar{p}\left(0\right) & =2\int_{0}^{a^{⋆}}\tau \left(a\right)\bar{p}\left(a\right)da,\\ \frac{d{\bar{x}}_{1}}{da} & =k_{1s}(\frac{{\bar{g}}_{f}}{k_{gf}+{\bar{g}}_{f}})-k_{14}{\bar{x}}_{4}{\bar{x}}_{1}-k_{1d}(\frac{{\bar{x}}_{1}}{k_{1}+{\bar{x}}_{1}}),\\ \frac{d{\bar{x}}_{2}}{da} & =k_{21}(\frac{x_{2t}-{\bar{x}}_{2}}{k_{2}+\left(x_{2t}-{\bar{x}}_{2}\right)}){\bar{x}}_{1}-k_{32}{\bar{x}}_{2}{\bar{x}}_{3}-k_{2d}{\bar{x}}_{2},\\ \frac{d{\bar{x}}_{3}}{da} & =k_{3s}-k_{32}{\bar{x}}_{2}{\bar{x}}_{3}-k_{31}(\frac{{\bar{x}}_{3}}{k_{3}+{\bar{x}}_{3}}){\bar{x}}_{2}-k_{3d}{\bar{x}}_{3},\\ \frac{d{\bar{x}}_{4}}{da} & =k_{4s}+k_{42}(\frac{k_{34}}{k_{34}+{\bar{x}}_{3}}){\bar{x}}_{2}-k_{41}(\frac{{\bar{x}}_{4}}{k_{4}+{\bar{x}}_{4}}){\bar{x}}_{1}-k_{4d}{\bar{x}}_{4}, \end{array}\right. \end{array}$$ (22)

where $\bar{\gamma }=\gamma \left(\bar{N}\right)$, ${\bar{g}}_{f}=g_{f}\left(\bar{N}\right)$ and $\bar{N}=\int_{0}^{a^{⋆}}\left(\bar{q}\left(a\right)+\bar{p}\left(a\right)\right)da$. Since the ODEs of the cell-cycle model are age-dependent and with the input of growth factors at a steady-state, all cell-cycle states acquire a steady-state. Therefore, to investigate the steady-states of proliferating and quiescent cell populations $\bar{p}\left(a\right)$ and $\bar{q}\left(a\right)$, we do not need to solve the ODEs of the cell-cycle model explicitly. Consequently, solving the system (22) for $\bar{p}$ and $\bar{q}$, we obtain $\bar{q}$ as

$$\begin{array}{c} \bar{q}\left(a\right)=\frac{\chi \left(a,{\bar{x}}_{1}\right)\bar{p}\left(a\right)}{\bar{\gamma }+\mu_{q}\left(a\right)}, \end{array}$$ (23)

and after using the above relation for $\bar{q}$ in the equation for $\bar{p}$ yields

$$\begin{array}{c} \frac{d(g\left(a\right)\bar{p}\left(a\right))}{da}+(\frac{\chi \left(a,{\bar{x}}_{1}\right)\mu_{q}\left(a\right)}{\bar{\gamma }+\mu_{q}\left(a\right)}+\tau \left(a\right)+\mu_{p}\left(a\right))\bar{p}\left(a\right)=0. \end{array}$$ (24)

Solving Eq (24) for $\bar{p}\left(a\right)$, yields both steady-state solutions for $\bar{p}\left(a\right)$ and $\bar{q}\left(a\right)$ as follows

$$\begin{array}{c} \left\{ \begin{array}{cc} \bar{q}\left(a\right) & =\frac{\chi \left(a,{\bar{x}}_{1}\right)\bar{p}\left(0\right)}{\bar{\gamma }+\mu_{q}\left(a\right)}\text{exp}(-\int_{0}^{a}\frac{1}{g(a)}(g^{\prime }\left(a\right)+\frac{\chi \left({\bar{x}}_{1},\xi \right)\mu_{q}\left(\xi \right)}{\bar{\gamma }+\mu_{q}\left(\xi \right)}+\tau \left(\xi \right)+\mu_{p}\left(\xi \right))d\xi ),\\ \bar{p}\left(a\right) & =\bar{p}\left(0\right)\text{exp}(-\int_{0}^{a}\frac{1}{g(a)}(g^{\prime }\left(a\right)+\frac{\chi \left({\bar{x}}_{1},\xi \right)\mu_{q}\left(\xi \right)}{\bar{\gamma }+\mu_{q}\left(\xi \right)}+\tau \left(\xi \right)+\mu_{p}\left(\xi \right))d\xi ). \end{array}\right. \end{array}$$

It is clear that the system defined in Eqs (1) and (2), (10a)–(10d) always admits a trivial steady-state.

### 4.2 Stability analysis of steady-state solutions

Next, we want to derive the stability conditions for a positive steady-state solution. Suppose $q\left(a,t\right)=\bar{q}$ and $p\left(a,t\right)=\bar{p}$, ∀*t* ≥ 0 represent equilibrium solutions to the PDE model (1) and (2) and q*(*a*, *t*) and p*(*a*, *t*) represent the corresponding perturbation terms:

$$q\left(a,t\right)=\bar{q}+\epsilon q^{*}\left(a,t\right),\;p\left(a,t\right)=\bar{p}+\epsilon p^{*}\left(a,t\right).$$

Substituting the above relations in to the PDE model (1) and (2), we have

$$\begin{array}{c} \left\{ \begin{array}{cc} & \epsilon \frac{\partial }{\partial t}q^{*}\left(a,t\right)=\chi \left(a,{\bar{x}}_{1}\right)\left(\bar{p}+\epsilon p^{*}\left(a,t\right)\right)-(\frac{\nu \theta^{\kappa }}{\theta^{\kappa }+{\left(\bar{N}+\epsilon n\left(t\right)\right)}^{\kappa }}+\mu_{q}\left(a\right))\left(\bar{q}+\epsilon q^{*}\left(a,t\right)\right),\\ & \epsilon \frac{\partial }{\partial t}p^{*}\left(a,t\right)+\frac{\partial }{\partial a}\left(g\left(a\right)\left(\bar{p}+\epsilon p^{*}\left(a,t\right)\right)\right)=(\frac{\nu \theta^{\kappa }}{\theta^{\kappa }+{\left(\bar{N}+\epsilon n\left(t\right)\right)}^{\kappa }})\left(\bar{q}+\epsilon q^{*}\left(a,t\right)\right)\\ & \;-\left(\tau \left(a\right)+\chi \left(a,{\bar{x}}_{1}\right)+\mu_{p}\left(a\right)\right)\left(\bar{p}+\epsilon p^{*}\left(a,t\right)\right),\\ & \left(\bar{p}\left(0\right)+\epsilon p^{*}\left(0,t\right)\right)=2\int_{0}^{a^{⋆}}\tau \left(a\right)\left(\bar{p}+\epsilon p^{*}\left(a,t\right)\right)da. \end{array}\right. \end{array}$$

where, $n\left(t\right)≔\int_{0}^{a^{⋆}}(p^{*}\left(a,t\right)+q^{*}\left(a,t\right))da$. Then, take the derivative wrt epsilon *ϵ*, leads to:

$$\begin{array}{c} \left\{ \begin{array}{cc} & \frac{\partial }{\partial t}q^{*}\left(a,t\right)=\chi \left(a,{\bar{x}}_{1}\right)p^{*}\left(a,t\right)-(\frac{\partial }{\partial \epsilon }(\frac{\nu \theta^{\kappa }\epsilon }{\theta^{\kappa }+{\left(\bar{N}+\epsilon n\left(t\right)\right)}^{\kappa }})-\mu_{q}\left(a\right))q^{*}\left(a,t\right),\\ & \frac{\partial }{\partial t}p^{*}\left(a,t\right)+\frac{\partial }{\partial a}\left(g\left(a\right)p^{*}\left(a,t\right)\right)=\frac{\partial }{\partial \epsilon }(\frac{\nu \theta^{\kappa }\epsilon }{\theta^{\kappa }+{\left(\bar{N}+\epsilon n\left(t\right)\right)}^{\kappa }})q^{*}\left(a,t\right)\\ & \;-\left(\tau \left(a\right)+\chi \left(a,{\bar{x}}_{1}\right)+\mu_{p}\left(a\right)\right)p^{*}\left(a,t\right),\\ & p^{*}\left(0,t\right)=2\int_{0}^{a^{⋆}}\tau \left(a\right)p^{*}\left(a,t\right)da, \end{array}\right. \end{array}$$

which simplifies to

$$\begin{array}{c} \left\{ \begin{array}{cc} & \frac{\partial }{\partial t}q^{*}\left(a,t\right)=\chi \left(a,{\bar{x}}_{1}\right)p^{*}\left(a,t\right)-(\nu \theta^{\kappa }(\frac{\theta^{\kappa }+{\left(\bar{N}+\epsilon n\left(t\right)\right)}^{\kappa }-\kappa \epsilon n\left(t\right){\left(\bar{N}+\epsilon n\left(t\right)\right)}^{\kappa -1}}{{\left(\theta^{\kappa }+{\left(\bar{N}+\epsilon n\left(t\right)\right)}^{\kappa }\right)}^{2}})\\ & \;-\mu_{q}\left(a\right))q^{*}\left(a,t\right),\\ & \frac{\partial }{\partial t}p^{*}\left(a,t\right)+\frac{\partial }{\partial a}\left(g\left(a\right)p^{*}\left(a,t\right)\right)=\nu \theta^{\kappa }(\frac{\theta^{\kappa }+{\left(\bar{N}+\epsilon n\left(t\right)\right)}^{\kappa }-\kappa \epsilon n\left(t\right){\left(\bar{N}+\epsilon n\left(t\right)\right)}^{\kappa -1}}{{\left(\theta^{\kappa }+{\left(\bar{N}+\epsilon n\left(t\right)\right)}^{\kappa }\right)}^{2}})\\ & \;q^{*}\left(a,t\right)-\left(\chi \left(a,{\bar{x}}_{1}\right)+\tau \left(a\right)+\mu_{p}\left(a\right)\right)p^{*}\left(a,t\right),\\ & p^{*}\left(0,t\right)=2\int_{0}^{a^{⋆}}\tau \left(a\right)p^{*}\left(a,t\right)da. \end{array}\right. \end{array}$$

Taking the limit *ϵ* → 0, we obtain a linear system of PDEs:

$$\begin{array}{c} \;\{\begin{array}{cc} & q_{t}^{*}\left(a,t\right)=\chi \left(a,{\bar{x}}_{1}\right)p^{*}\left(a,t\right)-(\mu_{q}\left(a\right)+\gamma \left(\bar{N}\right))q^{*}\left(a,t\right),\\ & p_{t}^{*}\left(a,t\right)+\partial_{a}\left(g\left(a\right)p^{*}\left(a,t\right)\right)=\gamma \left(\bar{N}\right)q^{*}\left(a,t\right))-\left(\chi \left(a,{\bar{x}}_{1}\right)+\tau \left(a\right)+\mu_{p}\left(a\right)\right)p^{*}\left(a,t\right),\\ & p^{*}\left(0,t\right)=2\int_{0}^{a^{⋆}}\tau \left(a\right)p^{*}\left(a,t\right)da, \end{array}\; \end{array}$$ (25)

where $\gamma \left(\bar{N}\right)=\nu \theta^{\kappa }/\left(\theta^{\kappa }+{\bar{N}}^{\kappa }\right)$. Next, we formulate (25) as semilinear problem:

$$\begin{array}{c} \frac{d}{dt}\omega \left(t\right)=C\omega \left(t\right),\;\omega \left(0\right)=\omega_{0}\in X, \end{array}$$ (26)

on the Banach space *X* and the generator C is defined by

$$\begin{array}{c} \left(C\phi \right)\left(a\right)=(\begin{array}{c} -\left(\gamma \left(\bar{N}\right)+\mu_{q}\left(a\right)\right)\varphi_{1}\left(a\right)+\chi \left(a,{\bar{x}}_{1}\right)\varphi_{2}\left(a\right)\\ \gamma \left(\bar{N}\right)\varphi_{1}\left(a\right)-(\frac{\partial }{\partial a}+\frac{1}{g(a)}(\tau \left(a\right)+\chi \left(a,{\bar{x}}_{1}\right)+\mu_{p}\left(a\right)))g\left(a\right)\varphi_{2}\left(a\right) \end{array}), \end{array}$$

where

$$\phi \left(a\right)={\left(\phi_{1}\left(a\right),\phi_{2}\left(a\right)\right)}^{T}\in D\left(C\right),$$

where, D(C) is defined below:

$$D\left(C\right)=\{\left(\phi_{1},\phi_{2}\right)\;|\;\phi_{i}\;\;\text{is}\;\text{absolute}\;\text{continuous}\;\text{on}\;\left[0,a^{⋆}\right),\phi \left(0\right)={\left(2\int_{0}^{a^{⋆}}\tau \left(a\right)\phi_{2}\left(a\right)da,0\right)}^{T}\}.$$

Next, the resolvent equation for operator C is considered as,

$$\begin{array}{c} (\lambda I-C)\phi =\psi ,\;\phi \in D(C),\;\psi \in X,\;\lambda \in C. \end{array}$$ (27)

Which leads to

$$\begin{array}{cc} \;(\lambda +\gamma \left(\bar{N}\right)+\mu_{q}\left(a\right))\phi_{1}\left(a\right)-\chi \left(a,{\bar{x}}_{1}\right)\phi_{2}\left(a\right) & =\psi_{1}\left(a\right) \end{array}$$ (28a)

$$\begin{array}{cc} \;-\gamma \left(\bar{N}\right)\phi_{1}\left(a\right)+\frac{\partial }{\partial a}\left(g\left(a\right)\phi_{2}\left(a\right)\right)+(\lambda +\tau \left(a\right)+\chi \left(a,{\bar{x}}_{1}\right)+\mu_{p}\left(a\right))\phi_{2}\left(a\right) & =\psi_{2}\left(a\right), \end{array}$$ (28b)

and

$$\phi_{2}\left(0\right)=2\int_{0}^{a^{⋆}}\tau \left(a\right)\phi_{2}\left(a\right)da.$$

By solving (28a), we get

$$\begin{array}{c} \phi_{1}\left(a\right)=\frac{\psi_{1}\left(a\right)+\chi \left(a,{\bar{x}}_{1}\right)\phi_{2}\left(a\right)}{\lambda +\gamma \left(\bar{N}\right)+\mu_{q}\left(a\right)}. \end{array}$$ (29)

Which after substituting in Eq (28b) and solving gives

$$\begin{array}{cc} \phi_{2}\left(a\right)= & \text{exp}(-\int_{0}^{a}\tau \left(\xi \right)+\chi \left({\bar{x}}_{1},\xi \right)+\lambda +\mu_{p}\left(\xi \right)-\frac{\gamma \left(\bar{N}\right)\chi \left({\bar{x}}_{1},\xi \right)}{g\left(\xi \right)(\lambda +\gamma \left(\bar{N}\right)+\mu_{q}\left(\xi \right))}d\xi )\\ & \left[\int_{0}^{a}\text{exp}(\int_{0}^{\zeta }\tau \left(\xi \right)+\chi \left({\bar{x}}_{1},\xi \right)+\lambda +\mu_{p}\left(\xi \right)-\frac{\gamma \left(\bar{N}\right)\chi \left({\bar{x}}_{1},\xi \right)}{g\left(\xi \right)(\lambda +\gamma \left(\bar{N}\right)+\mu_{q}\left(\xi \right))}d\xi \right)\\ & \frac{1}{g(\zeta )}\{\psi_{2}\left(\zeta \right)+\frac{\gamma \left(\bar{N}\right)\psi_{1}\left(\zeta \right)}{\lambda +\gamma \left(\bar{N}\right)+\mu_{q}\left(\zeta \right)}\}d\zeta +\phi_{2}\left(0\right)]. \end{array}$$

Substituting *ϕ*_2_(*a*) back in Eq (29) yields


$$\begin{array}{cc} \phi_{1}\left(a\right)= & \frac{1}{\lambda +\gamma \left(\bar{N}\right)+\mu_{q}\left(a\right)}[\text{exp}(-\int_{0}^{a}\tau \left(\xi \right)+\chi \left({\bar{x}}_{1},\xi \right)+\lambda +\mu_{p}\left(\xi \right)\\ & -\frac{\gamma \left(\bar{N}\right)\chi \left({\bar{x}}_{1},\xi \right)}{g\left(\xi \right)(\lambda +\gamma \left(\bar{N}\right)+\mu_{q}\left(\xi \right))}d\xi )\{\int_{0}^{a}\text{exp}(\int_{0}^{\zeta }\tau \left(\xi \right)+\chi \left({\bar{x}}_{1},\xi \right)+\lambda +\mu_{p}\left(\xi \right)\\ & -\frac{\gamma \left(\bar{N}\right)\chi \left({\bar{x}}_{1},\xi \right)}{g\left(\xi \right)(\lambda +\gamma \left(\bar{N}\right)+\mu_{q}\left(\xi \right))}d\xi )\frac{1}{g(\zeta )}\{\psi_{2}\left(\zeta \right)+\frac{\gamma \left(\bar{N}\right)\psi_{1}\left(\zeta \right)}{\lambda +\gamma \left(\bar{N}\right)+\mu_{q}\left(\zeta \right)}\}d\zeta \\ & +\phi_{2}\left(0\right)\}\chi \left(a,{\bar{x}}_{1}\right)+\psi_{1}\left(a\right)]. \end{array}$$

**Lemma 4.1**. *The operator C has a compact resolvent and*

$$\begin{array}{c} \sigma \left(C\right)=\sigma_{P}\left(C\right)=\left\{\lambda \in C\;|-\mu_{q}-\gamma \left(\bar{N}\right)\in \sigma_{p}\left(U_{\lambda }\right)\right\}, \end{array}$$ (30)

*where* *σ*(C) *is the spectrum and* *σ*_*P*_(C) *represents the point spectrum of operator* C.

*Proof*. Let’s rewrite *ϕ*_1_(*a*) as

$$\begin{array}{c} \phi_{1}\left(a\right)=\left(U_{\lambda }\psi_{2}\right)\left(a\right)+\left(V_{\lambda }\psi_{1}\right)\left(a\right), \end{array}$$

where *U*_λ_ and *V*_λ_ are the linear operators on Banach space, given as

$$\begin{array}{c} \left(U_{\lambda }\psi \right)\left(a\right)=\int_{0}^{a^{⋆}}G_{\lambda }\left(\zeta ,a\right)\psi \left(\zeta \right)d\zeta ,\;\;\left(V_{\lambda }\psi \right)\left(a\right)=\int_{0}^{a^{⋆}}H_{\lambda }\left(\zeta ,a\right)\psi \left(\zeta \right)d\zeta , \end{array}$$ (31)

where

$$\begin{array}{cc} G_{\lambda }\left(\zeta ,a\right)= & \frac{\chi (a,{\bar{x}}_{1})}{g\left(\zeta \right)(\lambda +\gamma \left(\bar{N}\right)+\mu_{q}\left(a\right))}\text{exp}(-\int_{0}^{a}\tau \left(\xi \right)+\chi \left({\bar{x}}_{1},\xi \right)+\lambda +\mu_{p}\left(\xi \right)\\ & -\frac{\gamma \left(\bar{N}\right)\chi \left({\bar{x}}_{1},\xi \right)}{g\left(\xi \right)(\lambda +\gamma \left(\bar{N}\right)+\mu_{q}\left(\xi \right))}d\xi )\text{exp}(\int_{0}^{\zeta }\tau \left(\xi \right)+\chi \left({\bar{x}}_{1},\xi \right)+\lambda +\mu_{p}\left(\xi \right)\\ & -\frac{\gamma \left(\bar{N}\right)\chi \left({\bar{x}}_{1},\xi \right)}{g\left(\xi \right)(\lambda +\gamma \left(\bar{N}\right)+\mu_{q}\left(\xi \right))}d\xi ), \end{array}$$ (32)

and

$$\begin{array}{cc} H_{\lambda }\left(\zeta ,a\right)= & \frac{1}{g\left(\zeta \right)(\lambda +\gamma \left(\bar{N}\right)+\mu_{q}\left(\xi \right))}(\gamma \left(\bar{N}\right)G_{\lambda }\left(\zeta ,a\right)+\frac{g(\zeta )}{a^{⋆}}). \end{array}$$

Similarly, we rewrite *ϕ*_2_(*a*) as

$$\begin{array}{c} \phi_{2}\left(a\right)=\frac{1}{\chi (a,{\bar{x}}_{1})}\{\left(\lambda +\gamma \left(\bar{N}\right)+\mu_{q}\right)\left(U_{\lambda }\psi_{2}\right)\left(a\right)+\gamma (\bar{N})\left(U_{\lambda }\psi_{1}\right)\left(a\right)\}. \end{array}$$

Let $\Lambda =\{\lambda \in C\;|-\mu_{q}\left(\cdot \right)-\gamma \left(\bar{N}\right)\in \sigma \left(U_{\lambda }\right)\}$, then we can say that if λ∈C\Λ, operators *U*_λ_ and *V*_λ_ are compact operators from *X* to *L*^1^(0, *a*^⋆^). This implies *ϕ*_1_(*a*) is represented by a compact operator. In a similar fashion, *ϕ*_2_(*a*) is also represented by a compact operator. Resultantly, we get that operator C has a compact resolvent which further implies that *σ*(C) comprises entirely of isolated eigenvalues, i.e., *σ*(C) = *σ*_*P*_(C) (see p. 187, Theorem 6.29 in [46]). From latter, we know that C\Λ⊂ρ(C), where *ρ*(C) is the resolvent of C. This implies *σ*_*P*_(C) = *σ*(C) ⊂ Λ. Since *U*_λ_ is a compact operator, then it leads to *σ*(*U*_λ_)\{0} = *σ*_*P*_(*U*_λ_)\{0}. Now if λ ∈ Λ, there exists an eigenfunction *ψ*_λ_ such that *U*_λ_*ψ*_λ_ = *ψ*_λ_. Then, it is trivial to see that (*ϕ*_1_(*a*), *ϕ*_2_(*a*))^T^ provides an eigenvector of C for an eigenvalue λ. Then Λ ⊂ *σ*_*P*_(C), and finally, we can say that (30) satisfies.

**Lemma 4.2**. *Let* **T**(*t*) *be the* *C*_0_-*semigroup generated by the operator* C, *t* ≥ 0. *Then*, **T**(*t*) *is eventually norm continuous (ENC) and*

$$\begin{array}{c} \omega_{0}\left(C\right)=s\left(C\right)=\text{sup}\left\{\text{Re}\lambda \;|\lambda \in \sigma \left(C\right)\right\}, \end{array}$$ (33)

*where* *ω*_0_(C) *represents the growth bound of semigroup* **T**(*t*) *and* *s*(C) *denotes the spectral bound of the operator* C.

*Proof*. First, we write the bounded operator C as:

$$\begin{array}{c} C\phi =(\begin{array}{cc} -(\gamma \left(\bar{N}\right)+\mu_{q}\left(a\right)) & \chi (a,{\bar{x}}_{1})\\ \gamma (\bar{N}) & -(\frac{\partial }{\partial a}+\frac{1}{g(a)}\left(\tau \left(a\right)+\chi \left(a,{\bar{x}}_{1}\right)+\mu_{p}\left(a\right)\right))g\left(a\right) \end{array})(\begin{array}{c} \phi_{1}\left(a\right)\\ \phi_{2}\left(a\right) \end{array}), \end{array}$$

for *ϕ* ∈ *X*. To prove the compactness of C, we show that for any bounded sequence ${\left(\phi^{n}\right)}_{n\in N}$ in *X*, the sequence ${\left(C\phi^{n}\right)}_{n\in N}$ has a uniformly convergent subsequence. For this we use the Arzelà-Ascoli Theorem. Thereby, we need to check that ${\left(C\phi^{n}\right)}_{n\in N}$ is uniformly bounded and uniformly equicontinuous. For the boundedness, note that since we assumed that ${\left(\phi^{n}\right)}_{n\in N}$ is bounded, we have

$$\|C\phi^{n}{\|}_{1}\leq \left\|C\|\;\right\|\phi^{n}{\|}_{1}\leq \|C\|\;\underset{n\in N}{\text{sup}}{\left\|\phi^{n}\right\|}_{1},$$

proving that ${\left(C\phi^{n}\right)}_{n\in N}$ is also bounded. Next, for the uniform equicontinuity, consider

$$\begin{array}{cc} \int_{R} & |\left(C\phi \right)\left(a+h\right)-\left(C\phi \right)\left(a\right)|da=\int_{R}|C\left(a+h\right)-C\left(a\right)|\;|\phi \left(a\right)|da\\ & \leq \;\int_{R}|\;(\begin{array}{cc} -\gamma \left(\bar{N}\right)-\mu_{q}\left(a+h\right) & \chi (a+h,{\bar{x}}_{1})\\ \gamma (\bar{N}) & -\frac{\partial }{\partial a+h}g\left(a+h\right)\;-\;\tau \left(a+h\right)\;-\;\chi \left(a+h,{\bar{x}}_{1}\right)\;-\;\mu_{p}\left(a+h\right)\; \end{array})\\ & -(\begin{array}{cc} -\gamma \left(\bar{N}\right)-\mu_{q}\left(a\right) & \chi (a,{\bar{x}}_{1})\\ \gamma (\bar{N}) & -\frac{\partial }{\partial a}g\left(a\right)-\tau \left(a\right)-\chi \left(a,{\bar{x}}_{1}\right)-\mu_{p}\left(a\right) \end{array})||\begin{array}{c} \phi_{1}\left(a\right)\\ \phi_{2}\left(a\right) \end{array}|da\\ & =\int_{R}|\begin{array}{cc} -\mu_{q}\left(a+h\right)+\mu_{q}\left(a\right) & \chi \left({\bar{x}}_{1},a+h\right)-\chi \left(a,{\bar{x}}_{1}\right)\\ 0 & k(a+h)-k(a) \end{array}||\begin{array}{c} \phi_{1}\left(a\right)\\ \phi_{2}\left(a\right) \end{array}|da\\ & \leq \left\|\phi \right\|\int_{R}|\begin{array}{cc} -\mu_{q}\left(a+h\right)+\mu_{q}\left(a\right) & \chi \left({\bar{x}}_{1},a+h\right)-\chi \left(a,{\bar{x}}_{1}\right)\\ 0 & k(a+h)-k(a) \end{array}|da, \end{array}$$

where $k\left(a\right)=-\frac{\partial }{\partial a}g\left(a\right)-\tau \left(a\right)-\chi \left(a,{\bar{x}}_{1}\right)-\mu_{p}\left(a\right)$. It follows that ${\left(C\phi^{n}\right)}_{n\in N}$ is equicontinuous. Thus, by the Arzelà-Ascoli Theorem, the sequence ${\left(C\phi^{n}\right)}_{n\in N}$ has a uniformly convergent subsequence, and therefore, C is compact which implies **T** is ENC semigroup. As we know that the spectral mapping theorem applies to ENC semigroup, we get the spectral determined growth condition, i.e., *ω*_0_(C) = *s*(C), thus we obtain (33).

If *ω*_0_(C) < 0, the steady-state solution *ω* = 0 of (26) is locally exponentially asymptotically stable in a way that there exists *ϵ* > 0, *M* ≥ 1 and *γ* < 0, such that when *x* ∈ *X* and ‖*x*‖ ≤ *ϵ*, then the solution *ω*(*t*, *x*) of (26) exists globally and ‖*ω*(*t*, *x*)‖ ≤ *M* exp (*γt*)‖*x*‖, ∀*t* > 0.

Next, to study the stability of equilibrium states, we need to find that the dominant singular point, i.e., the element of set Λ which has the largest real part. Then utilizing (30) and (33), we can find the growth bound of semigroup **T**.

**Lemma 4.3**. *The operator* *U*_λ_, λ∈R *is nonsupporting with respect to* *X*_+_ *and*

$$\begin{array}{c} \underset{\lambda \to +\infty }{\text{lim}}r\left(U_{\lambda }\right)=0, \end{array}$$ (34)

*holds*.

*Proof*. It can be seen from (31) and (32) that the operator $U_{\lambda },\;\lambda \in R$ is strictly positive. Now, in order to show non-supporting property of $U_{\lambda },\;\lambda \in R$, we can easily verify the inequality

$$\begin{array}{c} U_{\lambda }\psi \geq \left\langle f_{\lambda },\psi \right\rangle c,\;c=1\in X_{+},\;\;\psi \in X_{+}, \end{array}$$ (35)

where the linear function *f*_λ_, is given as

$$\begin{array}{cc} \langle f_{\lambda },\psi \rangle \;=\; & \int_{0}^{a^{⋆}}\;[\frac{s(\zeta )}{g\left(\zeta \right)(\lambda +\gamma \left(\bar{N}\right)+\mu_{q}\left(a\right))}\text{exp}(\;-\;\int_{0}^{a}\tau \left(\xi \right)+\chi \left({\bar{x}}_{1},\xi \right)\;+\;\lambda \;+\;\mu_{p}\left(\xi \right)\\ & -\frac{\gamma \left(\bar{N}\right)\chi \left({\bar{x}}_{1},\xi \right)}{g\left(\xi \right)(\lambda +\gamma \left(\bar{N}\right)+\mu_{q}\left(\xi \right))}d\xi )\text{exp}(\int_{0}^{\zeta }\tau \left(\xi \right)+\chi \left({\bar{x}}_{1},\xi \right)+\lambda +\mu_{p}\left(\xi \right)\\ & -\frac{\gamma \left(\bar{N}\right)\chi \left({\bar{x}}_{1},\xi \right)}{g\left(\xi \right)(\lambda +\gamma \left(\bar{N}\right)+\mu_{q}\left(\xi \right))}d\xi )]\psi \left(\zeta \right)d\zeta . \end{array}$$ (36)

Thereby, it leads us to $U_{\lambda }^{n+1}\psi \geq \langle f_{\lambda },\psi \rangle {\langle f_{\lambda },c\rangle }^{n}c,\;\forall n.$ Since *f*_λ_ is strictly positive and the constant function *c* = 1 is a quasi-interior point of *L*^1^(0, *a*^⋆^), it leads to $\langle F,U_{\lambda }^{n}\rangle >0$ for every pair *ψ* ∈ *X*_+_\{0}, $F\in X_{+}^{*}\backslash \left\{0\right\}$. Then $U_{\lambda },\;\lambda \in R$ is nonsupporting. Following that, we utilise (35) and take duality pairing with the eigenfunctional *F*_λ_ of *U*_λ_ which corresponds to *r*(*U*_λ_), then we get

$$r\left(U_{\lambda }\right)\left\langle F_{\lambda },\psi \right\rangle \geq \left\langle F_{\lambda },e\right\rangle \left\langle f_{\lambda },\psi \right\rangle .$$

Suppose *ψ* = *c*, we obtain an inequality *r* (*U*_λ_) ≥ 〈*f*_λ_, *c*〉, where

$$\begin{array}{cc} \langle f_{\lambda },c\rangle = & \int_{0}^{a^{⋆}}\;\frac{s(\zeta )}{g\left(\zeta \right)(\lambda +\gamma \left(\bar{N}\right)+\mu_{q}\left(\zeta \right))}\text{exp}(\;-\;\int_{0}^{a}\tau \left(\xi \right)+\chi \left({\bar{x}}_{1},\xi \right)+\lambda +\mu_{p}\left(\xi \right)\\ & -\frac{\gamma \left(\bar{N}\right)\chi \left({\bar{x}}_{1},\xi \right)}{g\left(\xi \right)(\lambda +\gamma \left(\bar{N}\right)+\mu_{q}\left(\xi \right))}d\xi )\text{exp}(\int_{0}^{\zeta }\tau \left(\xi \right)+\chi \left({\bar{x}}_{1},\xi \right)+\lambda +\mu_{p}\left(\xi \right)\\ & -\frac{\gamma \left(\bar{N}\right)\chi \left({\bar{x}}_{1},\xi \right)}{\lambda +\gamma \left(\bar{N}\right)+\mu_{q}\left(\xi \right)}d\xi )d\zeta . \end{array}$$ (37)

It follows that

$$\begin{array}{cc} \langle f_{\lambda },c\rangle \geq & \epsilon \int_{0}^{a^{⋆}}\frac{1}{g\left(\zeta \right)(\lambda +\gamma \left(\bar{N}\right)+\mu_{q}\left(\zeta \right))}\text{exp}(-\int_{0}^{a}\tau \left(\xi \right)+\chi \left({\bar{x}}_{1},\xi \right)+\lambda +\mu_{p}\left(\xi \right)\\ & -\frac{\gamma \left(\bar{N}\right)\chi \left({\bar{x}}_{1},\xi \right)}{\lambda +\gamma \left(\bar{N}\right)+\mu_{q}\left(\xi \right)}d\xi )\text{exp}(\int_{0}^{\zeta }\tau \left(\xi \right)+\chi \left({\bar{x}}_{1},\xi \right)+\lambda +\mu_{p}\left(\xi \right)\\ & -\frac{\gamma \left(\bar{N}\right)\chi \left({\bar{x}}_{1},\xi \right)}{\lambda +\gamma \left(\bar{N}\right)+\mu_{q}\left(\xi \right)}d\xi )d\zeta . \end{array}$$ (38)

By using the positivity of $\gamma \left(\bar{N}\right),\;\mu_{p},\;\mu_{q},\;\chi$ and *τ*, we conclude the following

$$\underset{\lambda \to +\infty }{\text{lim}}r\left(U_{\lambda }\right)=0.$$

Hence proved.

Preceding Lemma concludes that λ → *r*(*U*_λ_) is a decreasing function of λ∈R. Furthermore, if λ∈R so that *r*(*U*_λ_) = 1, then λ ∈ Λ since *r*(*U*_λ_) ∈ *σ*_*P*_(*U*_λ_). From the monotonicity of *r*(*U*_λ_) and (34), the following holds.

**Lemma 4.4**. *There exists a unique* $\lambda_{0}\in R\cap \Lambda$ *such that* $r(U_{\lambda_{0}})=1$, *and* λ_0_ > 0 *if* *r*(*U*_0_) > 1; λ_0_ = 0 *if* *r*(*U*_0_) = 1; λ_0_ < 0 *if* *r*(*U*_0_) < 1.

Now, we will show, using Theorem 6.13 in [47], that λ_0_ is a dominant singular point.

**Lemma 4.5**. *If there exists a* λ ∈ Λ, λ ≠ λ_0_, *then* *Re*λ < λ_0_.

*Proof*. Suppose that λ ∈ Λ and *U*_λ_*ψ* = *ψ*, then |*U*_λ_*ψ*| = |*ψ*|, where |*ψ*|(*a*) = |*ψ*(*a*)|. This yields *U*_Reλ_*ψ* ≥ *ψ*. Considering the duality pairing with $F_{\text{Re}\lambda }\in X_{+}^{⋆}$, we get *r*(*U*_Reλ_)〈*F*_Reλ_, |*ψ*|〉 ≥ 〈*F*_Reλ_, |*ψ*|〉, which results into the fact that *r*(*U*_Reλ_)≥1 since *F*_Reλ_ is strictly positive. As shown that $r(U_{\lambda }),\lambda \in R$ is declining function, it concludes that Reλ ≤ λ_0_. If we suppose that Reλ = λ_0_, then $U_{\lambda_{0}}\left|\psi \right|=\left|\psi \right|$. In fact, if we assume $U_{\lambda_{0}}\left|\psi |>|\psi \right|$ and take duality pairing with the eigenfunctional *F*_0_ corresponding to $r(U_{\lambda_{0}})=1$ on both sides results into 〈*F*_0_, |*ψ*|〉 > 〈*F*_0_, |*ψ*|〉, which is a contradiction. As a consequence $U_{\lambda_{0}}\left|\psi \right|=\left|\psi \right|$, from which we deduce that |*ψ*| = *cψ*_0_, where *c* is a constant which we may assume 1 and *ψ*_0_ is the eigenfunction corresponding to $r(U_{\lambda_{0}})=1$. Therefore, *ψ*(*a*) = *ψ*_0_(*a*)exp(*iv*(*a*)) for, say, a real-valued function *v*(*a*). Substituting which into $U_{\lambda_{0}}\psi_{0}=\left|U_{\lambda }\psi \right|$, leads us to

$$\begin{array}{cc} & \frac{\chi (a,{\bar{x}}_{1})}{g\left(a\right)(\lambda_{0}+\gamma \left(\bar{N}\right)+\mu_{q}\left(a\right))}\int_{0}^{a^{⋆}}\text{exp}(\int_{a}^{\zeta }\tau \left(\xi \right)+\chi \left({\bar{x}}_{1},\xi \right)+\lambda_{0}+\mu_{p}\left(\xi \right)\\ & -\frac{\gamma \left(\bar{N}\right)\chi \left({\bar{x}}_{1},\xi \right)}{\lambda_{0}+\gamma \left(\bar{N}\right)+\mu_{q}\left(\xi \right)}d\xi )\psi_{0}\left(\zeta \right)d\zeta \\ = & |\frac{\chi (a,{\bar{x}}_{1})}{g\left(a\right)(\lambda_{0}+i\text{Im}\lambda +\gamma \left(\bar{N}\right)+\mu_{q}\left(a\right))}\int_{0}^{a^{⋆}}\text{exp}(\int_{a}^{\zeta }\tau \left(\xi \right)+\chi \left({\bar{x}}_{1},\xi \right)+\lambda_{0}+i\text{Im}\lambda \\ & +\mu_{p}\left(\xi \right)-\frac{\gamma \left(\bar{N}\right)\chi \left({\bar{x}}_{1},\xi \right)}{\lambda_{0}+i\text{Im}\lambda +\gamma \left(\bar{N}\right)+\mu_{q}\left(\xi \right)}d\xi )\text{exp}\left(iv\left(\zeta \right)\right)\psi_{0}\left(\zeta \right)d\zeta |. \end{array}$$

From Lemma 6.12 [47], it leads us to Imλ+ *v*(*ζ*) = Θ, where Θ is a constant. Utilizing *U*_λ_*ψ* = *ψ*, we get

$$\text{exp}\left(i\Theta \right)U_{\lambda_{0}}\psi_{\lambda_{0}}=\psi_{\lambda_{0}}\text{exp}\left(iv\left(\zeta \right)\right),$$

so Θ = *v*(*ζ*), leads to Imλ = 0. Hence proved.

**Theorem 4.6**. *The equilibrium state* ${\left(\bar{q}\left(a\right),\bar{p}\left(a\right)\right)}^{T},$ *for* (1) and (2) *is locally asymptotically stable if* *r*(*U*_0_) < 1 *and locally unstable if* *r*(*U*_0_) > 1.

*Proof*. Lemma 4.4 and 4.5 concludes that $\text{sup}\left\{\text{Re}\lambda :-\mu_{q}-\gamma \left(\bar{N}\right)\in \sigma_{P}\left(U_{\lambda }\right)\right\}=\lambda_{0}$. Therefore, it results into $s\left(C\right)=\text{sup}\{\text{Re}\lambda :-\mu_{q}-\gamma \left(\bar{N}\right)\in \sigma_{P}\left(U_{\lambda }\right)\}<0$ if *r*(*U*_0_)<1 and *s*(C) > 0 if *r*(*U*_0_) > 1. Hence proved.

## 5 Results and discussion

This section presents some of the model simulation to understand the evolution of both sub-populations in relation with the cell-cycle dynamics. Table 3 shows the model parameters employed in the simulations. Most of the parameters are used from the literature, however the rest of them were unknown and we used some arbitrary values which are selected either likely to be biologically relevant or by using a range of values so that our numerical simulations exhibit the expected behavior of the model under implied assumptions. Please note that, we do not consider a specific organ while choosing parameters, instead we consider the parameters for stem cell lines and for experimental validation, one must identify these parameters for other cell lines. Furthermore, we assume that the maximal age *a*^⋆^ of the cells is 50. The spatial step size and the time step is used as Δ*a* = 0.5 and Δ*t* = 0.02, respectively. Additionally, for the sake of clarity, we also assume unit speed, i.e., *g*(*a*) = 1. Three case studies will be discussed in the following section.

**Table 3:** Parameters used in the simulations.

Parameter	Description	Value	Unit
*ν*	Maximum transition rate from quiescent to proliferation phase	0.3-0.8 [48, 49]	day^−1^
*θ*	Total cell population beyond which Γ is zero	0.095 × 10^6^ [49]	-
*κ*	Hill coefficient	1 [50]	-
*ρ*_1_	Maximum effect of Cyclin D-CDK4/6 on cell division	0.05-1 [48, 51]	day^−1^
*ρ*_2_	Value of Cyclin D-CDK4/6 complex to achieve half maximum effect	0.35	-
*γ*_1_	Hill coefficient	5-10 [49]	-
*σ*_1_	Maximum rate of switching cells from proliferating to quiescent phase	0.8-1 [49]	-
*σ*_2_	Switching Cyclin D-CDK4/6 complex value beyond which *χ* is close to zero	0.35	-
*σ*_3_	Switching age value beyond which *χ* is close to zero	15-18 [48, 49]	h
*γ*_2_	Hill coefficient	5-10 [49]	-
*γ*_3_	Hill coefficient	5-10 [49]	-
*k*_*t*_	Rate constant which measures the effect of total population on growth factors	1.80 × 10^−9^ [40]	-

### 5.1 Local stability of a non-trivial steady-state solution

**Fig 3:**
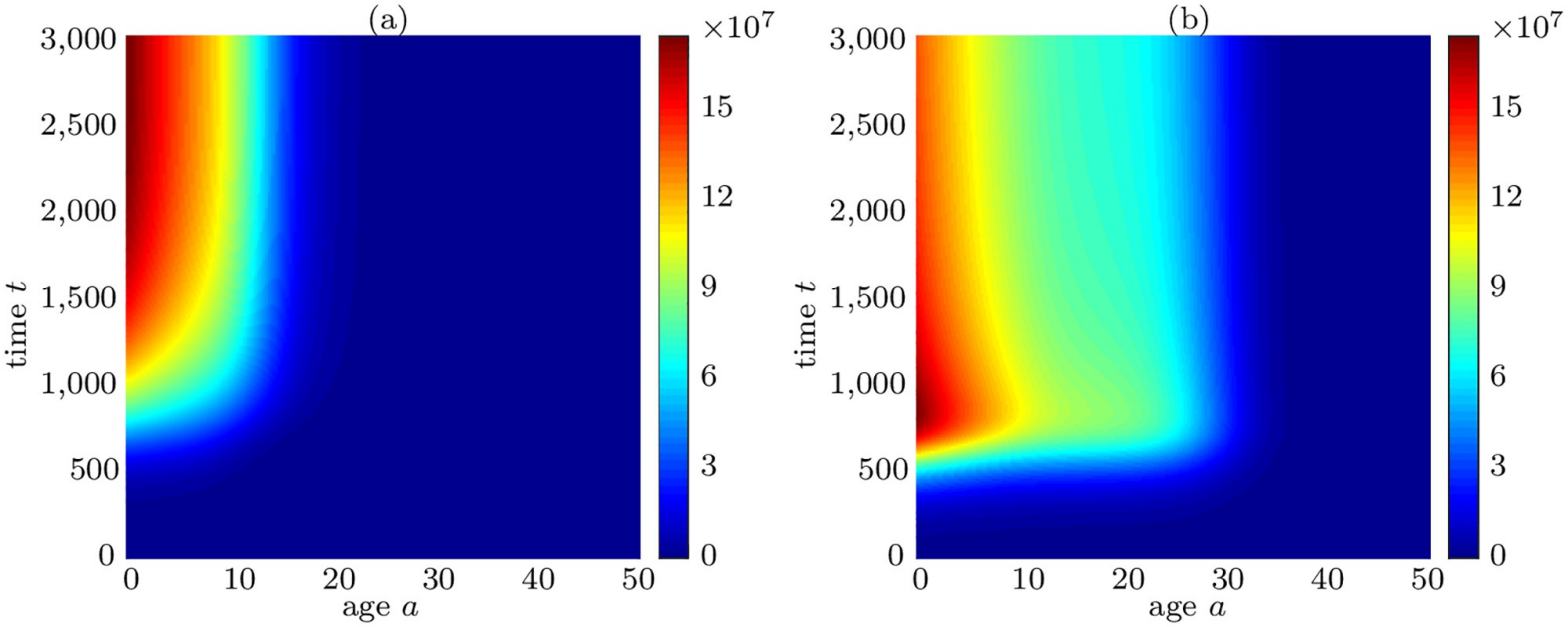
Cell number density distribution. (a) quiescent and (b) proliferating cell populations.

Here, we investigate local stability of the non-trivial equilibrium solution. The parameter values used are *μ*_*p*_ = *μ*_*q*_ = 0.0014. We take *γ*(*N*) = 6.8964 × 10^−6^ and *ρ*_1_ = 1.0. The initial conditions are assumed as $q\left(a,0\right)=p\left(a,0\right)=\frac{k_{0}}{\sqrt{2\pi \sigma^{2}}}\text{exp}(-\frac{{\left(a-\mu \right)}^{2}}{2\sigma^{2}})$, where *k*_0_ = 10^6^, *μ* = 2 and *σ*^2^ = 200. Fig 3 represents the number density distribution of quiescent *q*(*a*, *t*) (a) and the proliferating *p*(*a*, *t*) (b) cell populations. Both, quiescent and proliferating subpopulations evolve and achieve a steady-state over time.

**Fig 4:**
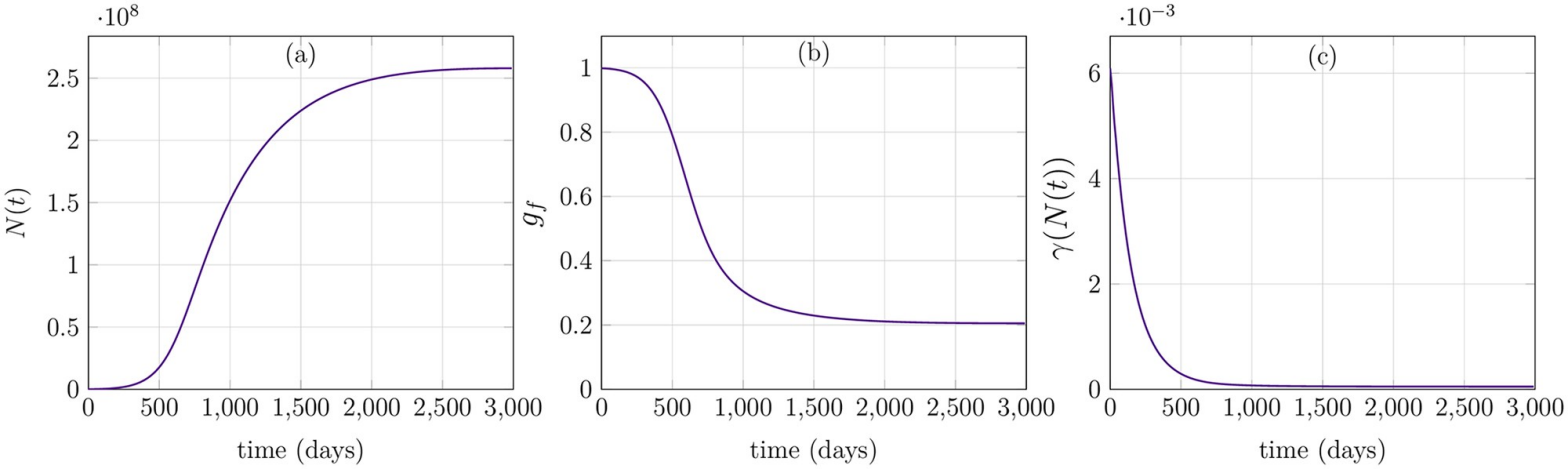
Behavior of total cell population, growth factors and gamma function. (a) *N*(*t*) achieves steady-state. (b) Growth-factors *g*_*f*_ decreasing with increase in cell population. (c) Gamma function *γ* declines as the total cell population achieves steady-state.

The overall cell population *N*(*t*), consisting of quiescent and proliferating cells, is increasing exponentially and eventually achieves a steady-state in Fig 4(a). Fig 4(b), on the other hand, demonstrates that the growth factors, which are regulated by cell population *N*(*t*), are maximum in the beginning because of low cell number and subsequently begin to decline until reaching an equilibrium. Finally, Fig 4(c) depicts transition rate of cells *γ*(*N*) to proliferating from quiescent phase. When the overall cell population increases, cell transition rate from quiescent to proliferating phase declines due to less growth factors. Since the population number is less and growth factors are maximal in the initial phase, (see Fig 4(a) and 4(b)), it leads to the activation and degradation of complex Cyclin D, which indicates a complete cell-cycle. Nevertheless, when growth factors decline to the level that only a few new cells are required, the complex Cyclin D does not show oscillatory dynamics and instead stays at reduced levels for all time, thus indicating no cell divisions. Here, a question may arise that how the behavior of a single cell can stand for the dynamics of whole population level. Indeed, the cell to cell variability aspect and spatial variance are dominating factors in this mechanism and predictions of our proposed model in Fig 3 are only representing an averaged behavior of all the cells in a population. The feedback signal itself in Eq (9) which depends on total cell population is an ideal representation of growth factors which entirely relies on total number of cells and ignores various other possible scenarios, for instance, availability of nutrients, PH level, oxygen concentration etc. Furthermore, the gamma function *γ*(*N*) which determines the cell transitions to proliferating from quiescent cells, is shown in Fig 4(c). It represents an inverse relation to total cell population and declines to a very low number when the respective cell populations attain a steady-state. In terms of feedback from cell-cycle to population level, merely Cyclin D-CDK4/6 complex’ concentration is taken into account. It mainly influences the transition rate *χ*(*a*, *x*_1_) from proliferating to quiescent cells. It is evident from the distribution of proliferating cells in Fig 3 that new cells are entering proliferating phase at age *a* = 0 and after 20 hours of aging, cells start leaving the proliferating phase depending on their cycle length and concentration of the complex Cyclin D-CDK4/6. However, quiescent cells *q*(*a*, *t*) are accumulating in the early proliferating phase which do not achieve certain threshold of Cyclin D-CDK4/6 concentration to pass through restriction point in the cell-cycle.

### 5.2 Local stability of the trivial solution

**Fig 5:**
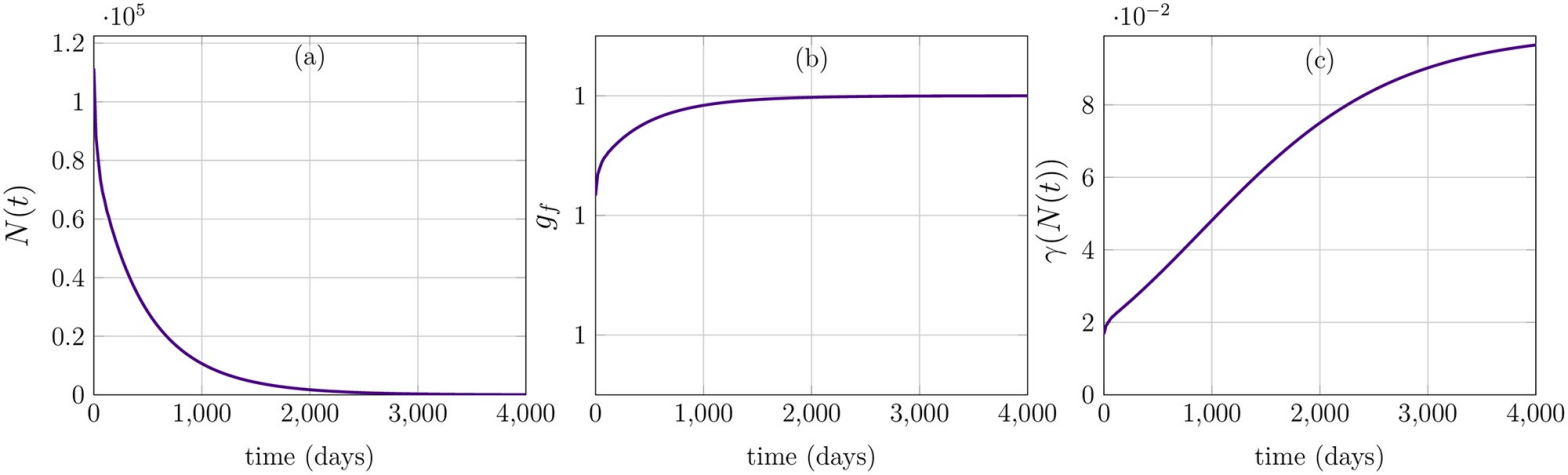
Trivial steady-state solution. (a) Total population of cells *N*(*t*) decays to zero. (b) Growth-factors *g*_*f*_ remain maximum due to decline in cell count. (c) Gamma function *γ* increasing to its maximum value due to less number of cell.

Next, we investigate the local stability of the trivial equilibrium solution. Thereby, we choose the death rates to be constants and *μ*_*p*_ = *μ*_*q*_ = 0.020. Moreover, we take *ρ*_1_ = 0.20 and *ν* = 0.1. We used the initial conditions as follows $p\left(a,0\right)=q\left(a,0\right)=\frac{k_{0}}{\sqrt{2\pi \sigma^{2}}}\text{exp}(-\frac{{\left(a-\mu \right)}^{2}}{2\sigma^{2}})$, where *k*_0_ = 10^6^, *μ* = 2 and *σ*^2^ = 200. The trivial equilibrium solution is locally stable as depicted in Fig 5. The parameters used in Fig 5 are same as mentioned above. The total cell population *N*(*t*) is plotted in Fig 5(a). The trivial steady-state is achieved until 2500 hours and cell population declines to zero. The growth factors, on the other hand, reach to their maximum value 1 and retain that value throughout due to very low cell number. The gamma function also attains its maximum with time.

### 5.3 Instability in the solution

**Fig 6:**
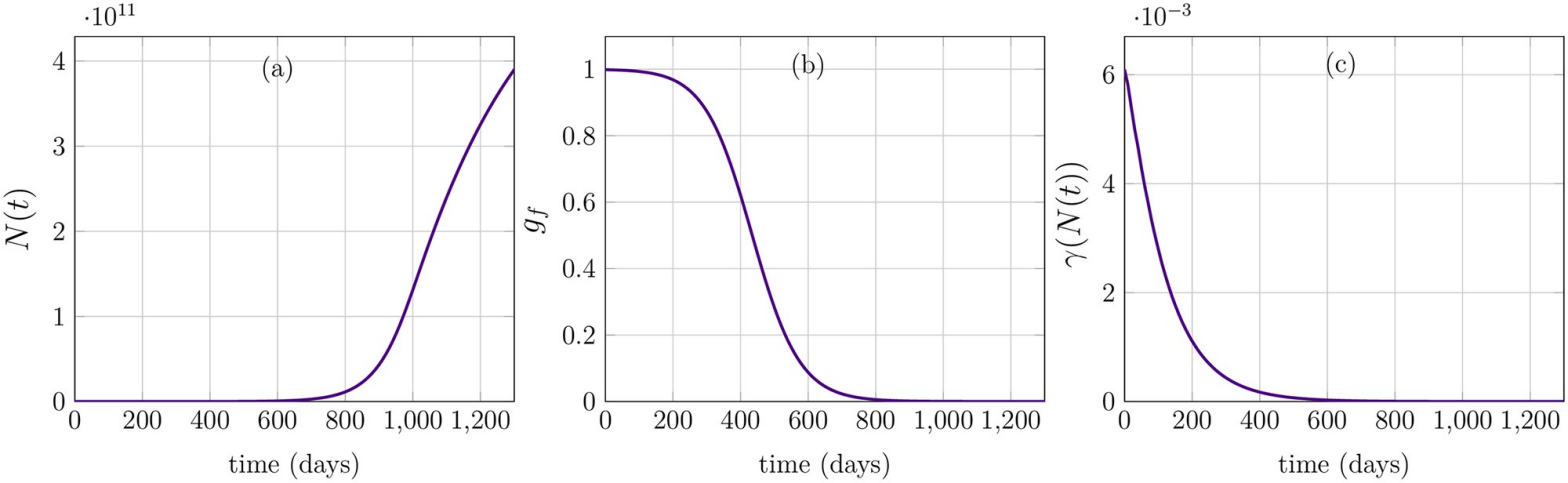
Unstable behavior. (a) Total cell population *N*(*t*) grows exponentially with time thus depicting an unstable behavior. (b) Growth-factors are increasing with rising total population of cells. However, as the change in *N*(*t*) larger and larger, the change in growth factors is negligible. (c) Gamma function is also declining.

Eventually, in Fig 6, we explore the parameters which lead to an instability in the solution. The proposed model, in general, displays very robust dynamics because of the closed feedback-loops. Nonetheless, transitioning function like *χ*(*a*, *x*_1_) display some sensitivity to the fluctuations in cell-cycle states. In order to pose a scenario to see abnormal cell-cycle behavior, we altered a parameter *k*_*gf*_ = 0.0001, which reflects a situation in which synthesis of Cyclin D-CDK4/6 complex is somewhat altered due to the change in the influence of growth factors on it. More precisely, by altering the parameter *k*_*gf*_, we induce delays in the oscillation of Cyclin D-CDK4/6 complex. Consequently, the cell number increases exponentially. Rest of the parameters are similar to other case studies explained before. Total cell population is plotted in Fig 6(a) which increases rapidly when there are abundant growth factors, see Fig 6(b). Finally, the transition function *γ*(*N*) is declining with time, as expected.

The proposed model does have some limitations also. The model, for example, excludes cell-to-cell variability, which is an important aspect to capture noise and heterogeneity from the cellular level. The feedback model, which includes growth factors, is relatively simple, and activation of the Cyclin D complex can only be characterized by taking into account all signaling pathways. Furthermore, at the microscale, the cell-cycle model is confined only to Cyclin D and the proteins in direct interaction with it; nevertheless, multiple additional proteins can control this network in various situations. Finally, while the Cyclin D complex and its inhibitor CDK4/6 plays a crucial part in the *G*_1_ to *S* phase transition, the other restriction point in the *S* phase for detecting DNA damage has been overlooked.

## 6 Conclusion

This study presents non-linear, multiscale modeling of physiologically-structured quiescent and proliferating cells in relation to cell-cycle dynamics, which play an essential part in committing a cell to irreversible cell-division process. We assume reversible transitioning from quiescent to proliferating cells and vice versa and, additionally, a feedback in both directions which maintains the homeostasis. We checked the wellposedness of the model, derive non-trivial equilibrium solutions and find spectral criteria for local stability in the sense that if the growth bound of the linearised semigroup is negative, the steady-state solution is the locally asymptotically stable, and if growth bound is positive, the steady-state solution is unstable. We also performed numerical simulations to study the behavior of the proposed model, and, in this regard, we studied three scenarios with some variation in the parameters. The first scenario explains the steady-state behavior of the model in any healthy person under normal conditions. The second scenario relates to a trivial steady-state where, hypothetically, the decline in cell number density is more than the rise due to newborn cells. Finally, in the third case study, we investigate the impact of Cyclin D-CDK4/6 complex on the transition between two sub-populations. It turns out that any fluctuations in synthesis and degradation of Cyclin D-CDK4/6 complex can result in an abnormal growth in cell number, thus leading to cancer. Moreover, it shows that the Cyclin complex plays a vital role in the reversible transition between the two subpopulations.

For possible future extensions of this work, we intend to extend the current modeling framework in an optimal control problem setting and to perform a thorough sensitivity analysis of the involved parameters.

## A Fréchet differentiability

**Lemma A.1**. *F*_1_: *X* → *X* *is Fréchet differentiable at* *ϕ* ∈ *X*, *where* *X* *is a Banach space*.

*Proof*. For *F*_1_ to be Fréchet differentiable, we need to show that

$$\begin{array}{c} \underset{\|h\|\to 0}{\text{lim}}\frac{\|F_{1}\left(\phi +h,\varphi \right)-F_{1}\left(\phi ,\varphi \right)-Ah\|}{\left\|h\right\|}=0, \end{array}$$ (39)

where *A* = *DF*_1_(*ϕ*) and ‖⋅‖ is a norm in *X*. Consider

$$\begin{array}{cc} & \frac{\|F_{1}\left(\phi +h,\varphi \right)-F_{1}\left(\phi ,\varphi \right)-Ah\|}{\left\|h\right\|}\\ = & \frac{1}{\left\|h\right\|}\|(\begin{array}{c} \frac{-\nu \theta^{\kappa }\left(\phi_{1}\left(a\right)+h\right)}{\theta^{\kappa }+{\left(N\phi +2a^{*}h\right)}^{\kappa }}+\alpha \left(\varphi_{1},a\right)\left(\phi_{2}\left(a\right)+h\right)\\ \frac{\nu \theta^{\kappa }\left(\phi_{1}\left(a\right)+h\right)}{\theta^{\kappa }+{\left(N\phi +2a^{*}h\right)}^{\kappa }}-\alpha \left(\varphi_{1},a\right)\left(\phi_{2}\left(a\right)+h\right) \end{array})-(\begin{array}{c} \frac{-\nu \theta^{\kappa }\phi_{1}\left(a\right)}{\theta^{\kappa }+{\left(N\phi \right)}^{\kappa }}+\alpha \left(\varphi_{1},a\right)\phi_{2}\left(a\right)\\ \frac{\nu \theta^{\kappa }\phi_{1}\left(a\right)}{\theta^{\kappa }+{\left(N\phi \right)}^{\kappa }}-\alpha \left(\varphi_{1},a\right)\phi_{2}\left(a\right) \end{array})\\ & -(\begin{array}{c} \frac{\partial }{\partial \phi_{1}}(\frac{-\nu \theta^{\kappa }\phi_{1}\left(a\right)}{\theta^{\kappa }+{\left(N\phi \right)}^{\kappa }}+\alpha \left(\varphi_{1},a\right)\phi_{2}\left(a\right))+\frac{\partial }{\partial \phi_{2}}(\frac{-\nu \theta^{\kappa }\phi_{1}\left(a\right)}{\theta^{\kappa }+{\left(N\phi \right)}^{\kappa }}+\alpha \left(\varphi_{1},a\right)\phi_{2}\left(a\right))\\ \frac{\partial }{\partial \phi_{1}}(\frac{\nu \theta^{\kappa }\phi_{1}\left(a\right)}{\theta^{\kappa }+{\left(N\phi \right)}^{\kappa }}-\alpha \left(\varphi_{1},a\right)\phi_{2}\left(a\right))+\frac{\partial }{\partial \phi_{2}}(\frac{\nu \theta^{\kappa }\phi_{1}\left(a\right)}{\theta^{\kappa }+{\left(N\phi \right)}^{\kappa }}-\alpha \left(\varphi_{1},a\right)\phi_{2}\left(a\right)) \end{array})h\|\\ = & \frac{1}{\left\|h\right\|}\|(\begin{array}{c} -\nu \theta^{\kappa }\phi_{1}\left(a\right)(\frac{1}{\theta^{\kappa }+{\left(N\phi +2a^{*}h\right)}^{\kappa }}-\frac{1}{\theta^{\kappa }+{\left(N\phi \right)}^{\kappa }})\\ \nu \theta^{\kappa }\phi_{1}\left(a\right)(\frac{1}{\theta^{\kappa }+{\left(N\phi +2a^{*}h\right)}^{\kappa }}-\frac{1}{\theta^{\kappa }+{\left(N\phi \right)}^{\kappa }}) \end{array})+(\begin{array}{c} \frac{-\nu \theta^{\kappa }}{\theta^{\kappa }+{\left(N\phi +2a^{*}h\right)}^{\kappa }}+\alpha \left(\varphi_{1},a\right)\\ \frac{\nu \theta^{\kappa }}{\theta^{\kappa }+{\left(N\phi +2a^{*}h\right)}^{\kappa }}-\alpha \left(\varphi_{1},a\right) \end{array})h\\ & -(\begin{array}{c} \frac{-\left(\theta^{\kappa }+{\left(N\phi \right)}^{\kappa }\right)\nu \theta^{\kappa }+\nu \theta^{\kappa }\phi_{1}\left(a\right)ka^{*}{\left(N\phi \right)}^{k-1}}{{\left(\theta^{\kappa }+{\left(N\phi \right)}^{\kappa }\right)}^{2}}+\frac{\nu \theta^{\kappa }\phi_{1}\left(a\right)ka^{*}{\left(N\phi \right)}^{k-1}}{{\left(\theta^{\kappa }+{\left(N\phi \right)}^{\kappa }\right)}^{2}}+\alpha \left(\varphi_{1},a\right)\\ \frac{\left(\theta^{\kappa }+{\left(N\phi \right)}^{\kappa }\right)\nu \theta^{\kappa }-\nu \theta^{\kappa }\phi_{1}\left(a\right)ka^{*}{\left(N\phi \right)}^{k-1}}{{\left(\theta^{\kappa }+{\left(N\phi \right)}^{\kappa }\right)}^{2}}-\frac{\nu \theta^{\kappa }\phi_{1}\left(a\right)ka^{*}{\left(N\phi \right)}^{k-1}}{{\left(\theta^{\kappa }+{\left(N\phi \right)}^{\kappa }\right)}^{2}}-\alpha \left(\varphi_{1},a\right) \end{array})h\|\\ = & \frac{1}{\left\|h\right\|}\|(\begin{array}{c} -\nu \theta^{\kappa }\phi_{1}\left(a\right)(\frac{{\left(N\phi \right)}^{\kappa }-{\left(N\phi +2a^{*}h\right)}^{\kappa }}{\left\{\theta^{\kappa }+{\left(N\phi +2a^{*}h\right)}^{\kappa }\right\}\left\{\theta^{\kappa }+{\left(N\phi \right)}^{\kappa }\right\}})\\ \nu \theta^{\kappa }\phi_{1}\left(a\right)(\frac{{\left(N\phi \right)}^{\kappa }-{\left(N\phi +2a^{*}h\right)}^{\kappa }}{\left\{\theta^{\kappa }+{\left(N\phi +2a^{*}h\right)}^{\kappa }\right\}\left\{\theta^{\kappa }+{\left(N\phi \right)}^{\kappa }\right\}}) \end{array})+(\begin{array}{c} \frac{-\nu \theta^{\kappa }}{\theta^{\kappa }+{\left(N\phi +2a^{*}h\right)}^{\kappa }}+\alpha \left(\varphi_{1},a\right)\\ \frac{\nu \theta^{\kappa }}{\theta^{\kappa }+{\left(N\phi +2a^{*}h\right)}^{\kappa }}-\alpha \left(\varphi_{1},a\right) \end{array})h\\ & -(\begin{array}{c} \frac{-\left(\theta^{\kappa }+{\left(N\phi \right)}^{\kappa }\right)+{\left(N\phi \right)}^{k-1}ka^{*}\phi_{1}\left(a\right)}{{\left(\theta^{\kappa }+{\left(N\phi \right)}^{\kappa }\right)}^{2}}+\frac{\phi_{1}\left(a\right)ka^{*}{\left(N\phi \right)}^{k-1}}{{\left(\theta^{\kappa }+{\left(N\phi \right)}^{\kappa }\right)}^{2}}+\frac{\alpha (\varphi_{1},a)}{\nu \theta^{\kappa }}\\ \frac{\left(\theta^{\kappa }+{\left(N\phi \right)}^{\kappa }\right)-{\left(N\phi \right)}^{k-1}ka^{*}\phi_{1}\left(a\right)}{{\left(\theta^{\kappa }+{\left(N\phi \right)}^{\kappa }\right)}^{2}}-\frac{\phi_{1}\left(a\right)ka^{*}{\left(N\phi \right)}^{k-1}}{{\left(\theta^{\kappa }+{\left(N\phi \right)}^{\kappa }\right)}^{2}}-\frac{\alpha (\varphi_{1},a)}{\nu \theta^{\kappa }} \end{array})\nu \theta^{\kappa }h\|\\ = & \|(\begin{array}{c} \frac{-1}{h}(\frac{{\left(N\phi \right)}^{\kappa }-{\left(N\phi +2a^{*}h\right)}^{\kappa }}{\left\{\theta^{\kappa }+{\left(N\phi +2a^{*}h\right)}^{\kappa }\right\}\left\{\theta^{\kappa }+{\left(N\phi \right)}^{\kappa }\right\}})\\ \frac{1}{h}(\frac{{\left(N\phi \right)}^{\kappa }-{\left(N\phi +2a^{*}h\right)}^{\kappa }}{\left\{\theta^{\kappa }+{\left(N\phi +2a^{*}h\right)}^{\kappa }\right\}\left\{\theta^{\kappa }+{\left(N\phi \right)}^{\kappa }\right\}}) \end{array})\nu \theta^{\kappa }\phi_{1}\left(a\right)+(\begin{array}{c} \frac{-\nu \theta^{\kappa }}{\theta^{\kappa }+{\left(N\phi +2a^{*}h\right)}^{\kappa }}+\alpha \left(\varphi_{1},a\right)\\ \frac{\nu \theta^{\kappa }}{\theta^{\kappa }+{\left(N\phi +2a^{*}h\right)}^{\kappa }}-\alpha \left(\varphi_{1},a\right) \end{array})\\ & -(\begin{array}{c} \frac{-\nu \theta^{\kappa }}{\theta^{\kappa }+{\left(N\phi \right)}^{\kappa }}+\alpha (\varphi_{1},a)\\ \frac{\nu \theta^{\kappa }}{\theta^{\kappa }+{\left(N\phi \right)}^{\kappa }}-\alpha (\varphi_{1},a) \end{array})\|. \end{array}$$ (40)

Further by taking the limit *h* → 0, we achieve

$$\begin{array}{c} \underset{\|h\|\to 0}{\text{lim}}\frac{\|F_{1}\left(\phi +h,\varphi \right)-F_{1}\left(\phi ,\varphi \right)-Ah\|}{\left\|h\right\|}=\|(\begin{array}{c} \frac{-\nu \theta^{\kappa }}{\theta^{\kappa }+{\left(N\phi \right)}^{\kappa }}+\alpha \left(\varphi_{1},a\right)\\ \frac{\nu \theta^{\kappa }}{\theta^{\kappa }+{\left(N\phi \right)}^{\kappa }}-\alpha \left(\varphi_{1},a\right) \end{array})-(\begin{array}{c} \frac{-\nu \theta^{\kappa }}{\theta^{\kappa }+{\left(N\phi \right)}^{\kappa }}+\alpha (\varphi_{1},a)\\ \frac{\nu \theta^{\kappa }}{\theta^{\kappa }+{\left(N\phi \right)}^{\kappa }}-\alpha (\varphi_{1},a) \end{array})\|=0. \end{array}$$ (41)

Thus, we show that *F*_1_ is Fréchet differentiable in *X*.

In the similar fashion, *F*_1_ and *F*_2_ can be shown Fréchet differentiable in both *X* and *Y*.
